# Supplementary material for: Social media use in adolescents with and without mental health conditions
Source: Nat Hum Behav. 2025 May 5;9(6):1283–99. doi: 10.1038/s41562-025-02134-4 (PMC12185317; doi:10.1038/s41562-025-02134-4)
Supplement: Supplementary file 1 — Supplementary Methods including Tables 1–4 and Figs. 1 and 2 and Results including Tables 5–22 and Figs. 3–5. [file 41562_2025_2134_MOESM1_ESM.pdf]

nature human behaviour

Supplementary information https://doi.org/10.1038/s41562-025-02134-4

Social media use in adolescents with and without mental health conditions

In the format provided by the authors and unedited

Table of Contents

Supplementary Methods (Stage 1) 3
Social media use variables 3
Supplementary Table 1. 3
Supplementary Table 2. 4
Mental health variables 5
Supplementary Table 3. 6
Supplementary Table 4. 7
Supplementary Figure 1. 9
Supplementary Figure 2. 10
Hypotheses and analysis plan 11
Choice of the smallest effect size of interest 11
Supplementary Design Table 14
Exploratory analyses for specific diagnoses or diagnostic categories 25
Supplementary results (Stage 2) 25
Descriptive statistics 25
Supplementary Table 5. 25
Supplementary Table 6. 28
Supplementary Table 7. 30
Supplementary Table 8. 32
Violin Plots 34
Supplementary Figure 3 34

Supplementary Figure 4 35
Supplementary Figure 5 36
Supplementary Table 9. 38
Non-parametric tests 39
Supplementary Table 10. 39
Supplementary Table 11. 41
Supplementary Table 12. 44
Supplementary Table 13. 45
Supplementary Table 14. 46
Sensitivity analyses 47
Between group comorbidity 47
Mental health severity 49
Individual diagnoses 50
Time spent on school days vs weekends 53
Power sensitivity analysis 54
Supplementary Table 22. 54

Supplementary Methods (Stage 1)

Social media use variables

Due to routing[1], the SMTimeSpent questions (weekday and weekend; SMTimeSpentS/SMTimeSpentw) were only asked of adolescents who responded to another survey question that they use social media (SMUse) and that their frequency of use (SMFreqofUse) is “daily or most days”. Adolescents who reported using social media sites between “a few times a week” to “less often than once a month” did not complete the SMTimeSpentS/W questions. Supplementary Table 1 provides an overview of the routing for the relevant social media questions, while supplementary Table 2 details our approach to handling missing data for the SMTimeSpent variables.

Supplementary Table 1.

Items in the MHCYP survey used to measure time spent on social media.

Variable	Use in analysis	Label	Answers	Routing
Social media use *(SMUse)*	No	Do you use any of the following social media sites or apps?	1 = AskFM; 2 = Bebo; 3 = Blogger; 4 = Facebook; 5 = Flickr; 6 = Google Hangouts and/or Google Plus (Google+); 7= Hi5; 8 = Instagram; 9 = Jabble; 10 = MySpace; 11= Piczo; 12 = Pinterest; 13 = SnapChat; 14 = Tumblr; 15 = Twitter; 16 = Vimeo; 17 =Vine; 18 = WhatsApp; 19 = YouTube; 20 = Skype; 21 = Musical.ly; 22 = Other; 23 =I do not use social media apps or sites.	None
Frequency of social media use (SMFreqofUse)	No	How often do you use social media sites or apps?	1 = Daily or most days; 2 = A few times a week; 3 = Once a week; 4 = A few times a month; 5 = Once a month; 6= Less often than once a month.	SMUse = 1 to 20, 22
Time spent on social media on weekdays (SMTimeSpentS)	Yes	When you use social media sites or apps how much time in total do you spend using them on a typical school day?	1 = Less than 30 minutes; 2 = More than 30 minutes but less than an hour; 3 = One to two hours 4 = Two to three hours; 5 = Three to four hours; 6 = Four to five hours; 7 = Five to six hours 8 = Six to seven hours; 9 = More than seven hours.	SMFreqofUse = 1

Time spent on social media on weekends (SMTimeSpentW)	Yes	When you use social media sites or apps how much time in total do you spend using them on a typical weekend or holiday day?	1 = Less than 30 minutes; 2 = More than 30 minutes but less than an hour; 3 = One to two hours 4 = Two to three hours; 5 = Three to four hours; 6 = Four to five hours; 7 = Five to six hours 8 = Six to seven hours; 9 = More than seven hours.	SMFreqofUse = 1

Supplementary Table 2.
Planned approach to missing data for the time spent items in the MHCYP survey.

**Variables**	**Missing values**	**Approach to missing data**
Total time spent (SMTimeSpentT; based on SMTimeSpentS and SMTimeSpentW)	Adolescents that responded to SMFreqofUse with anything other than 1 (Daily or most days) have missing values for SMTimeSpentS and SMTimeSpentW.	For people that answered 2-4 on SMFreqUse (2 = A few times a week; 3 = Once a week; 4 = A few times a month), we will input 45 minutes on the *SMTimeSpent* item (i.e., "more than 30 minutes but less than 1 hour").<br><br>For people that answered 5-6 on SMFreqofUse (5 = Once a month; 6 = Less often than once a month), we will input 15 minutes on the *SMTimeSpent* item (i.e., less than 30 minutes).

The questions capturing social media engagement were developed through consultation with a youth advisory board conducted by the National Center of Social Research in 2015. The board included 10 young people aged 16-24 years old, that were identified through existing mental health research networks and came from across England, with a wide range of experiences and interests. They all shared a common interest in mental health, linked to either first-lived experiences or involvement in the field.

Mental health variables

During the interview, participants completed the Strength and Difficulties Questionnaire (SDQ[2]) and the Development and Wellbeing Assessment (DAWBA[3]). The SDQ was administered first (Supplementary Table 4). This tool was used to increase the validity of the diagnostic assessment by allowing the identification of participants with mental health symptoms not picked up by the initial screening items on the DAWBA. Hence, if a participant scored highly on the SDQ, the interviewer was directed to ask in-depth questions from the DAWBA internalising modules.

The DAWBA uses structured and semi-structured questions to assess the presence and severity of symptoms for a wide range of DSM-5 or ICD-10 mental health conditions. Each condition is assigned a separate module. Each module starts with a few screening items (see Supplementary Table 4; e.g., "In the last 4 weeks, have there been times when you have been very sad, miserable, unhappy or tearful?"). If a participant responds positively to these structured items, they are subsequently asked unstructured questions about these problems (e.g., "Please describe your mood – sadness or irritability - and your level of interest in things").

During the assessment, interviewers transcribed unstructured responses on the DAWBA verbatim and were also able to add personal comments beneath each response. If a participant did not endorse one of the structured items (i.e., did not report mental health symptoms), the interview proceeded to the next module.

Having collected the information from respondents, the DAWBA online system then processes and presents the information to make it as easy as possible for trained clinical raters to decide if the child has a mental health condition. In Supplementary Figure 1, we provide a fictitious exemplar of a DAWBA module as shown to the clinical raters. Clinical raters can assign DSM-5 or ICD-10 mental health conditions based on the collected information. In Supplementary Figure 2, we provide a fictitious exemplar of the DAWBA overview completed by the clinical rater to assess the presence of a condition.

Supplementary Table 3.
Items from the Strength and Difficulties Questionnaire.

Item	Answer options		
I try to be nice to other people. I care about their feelings.	Not true	Somewhat true	Certainly true
I am restless, I cannot stay still for long.	Not true	Somewhat true	Certainly true
I get a lot of headaches, stomach-aches or sickness.	Not true	Somewhat true	Certainly true
I usually share with others (food, games, pens etc.).	Not true	Somewhat true	Certainly true
I get very angry and often lose my temper	Not true	Somewhat true	Certainly true
I am usually on my own. I generally play alone or keep to myself.	Not true	Somewhat true	Certainly true
I usually do as I am told.	Not true	Somewhat true	Certainly true
I worry a lot.	Not true	Somewhat true	Certainly true
I am helpful if someone is hurt, upset or feeling ill.	Not true	Somewhat true	Certainly true
I am constantly fidgeting or squirming.	Not true	Somewhat true	Certainly true
My attention is good.	Not true	Somewhat true	Certainly true
I have one good friend or more.	Not true	Somewhat true	Certainly true
I fight a lot. I can make other people do what I want.	Not true	Somewhat true	Certainly true
I am often unhappy, down-hearted or tearful.	Not true	Somewhat true	Certainly true
Other people my age generally like me	Not true	Somewhat true	Certainly true
I am easily distracted, I find it difficult to concentrate.	Not true	Somewhat true	Certainly true
I am nervous in new situations. I easily lose confidence.	Not true	Somewhat true	Certainly true
I am kind to younger children.	Not true	Somewhat true	Certainly true
I am often accused of lying or cheating.	Not true	Somewhat true	Certainly true
Other children or young people pick on me or bully me.	Not true	Somewhat true	Certainly true
I often volunteer to help others (parents, teachers, children).	Not true	Somewhat true	Certainly true
I think before I do things.	Not true	Somewhat true	Certainly true
I take things that are not mine from home, school or elsewhere.	Not true	Somewhat true	Certainly true
I get on better with adults than with people my own age.	Not true	Somewhat true	Certainly true

I have many fears, I am easily scared.	Not true	Somewhat true	Certainly true
I finish the work I'm doing.	Not true	Somewhat true	Certainly true

Supplementary Table 4.

Example of structured question from a DAWBA module on attention hyperactivity disorder.

DAWBA module	Example questions
Attention deficit hyperactivity disorder	11-19: Whether people outside the family complain about you having problems with overactivity or poor concentration?
	11-19: Whether your family complain about you having problems with overactivity or poor concentration
	11-19: Whether respondent thinks they have real problems with overactivity or poor concentration
	11-19: Thinking about the last 6 months: Do you often fidget?
	11-19: Thinking about the last 6 months: Is it hard for you to stay sitting down for long?
	11-19: Thinking about the last 6 months: Are you too full of energy and always on the go?
	11-19: Thinking about the last 6 months: Do you find it difficult to be quiet?
	11-19: Thinking about the last 6 months: Is it hard for you to unwind and relax?
	11-19: Thinking about the last 6 months: Do you often blurt out an answer before you have heard the question properly, or finish other people s sentences for them?
	11-19: Thinking about the last 6 months: Is it hard for you to wait your turn?
	11-19: Thinking about the last 6 months: Do you often interrupt other people when they are busy?

	11-19: Thinking about the last 6 months: Do you often go on talking even if you have been asked to stop, or if no one is listening?
	11-19: Thinking about the last 6 months: Do you often make careless mistakes or fail to pay attention to what you are supposed to be doing?
	11-19: Thinking about the last 6 months: Do you often lose interest in what you are doing?
	11-19: Thinking about the last 6 months: Do you often find it hard to concentrate on what other people are saying to you?
	11-19: Thinking about the last 6 months: Is it often hard for you finish a job properly, doing all the last bits after the most interesting bits have been done?
	11-19: Thinking about the last 6 months: Is it often hard for you to get yourself organized to do something?
	11-19: Thinking about the last 6 months: Do you often try to get out of things you would have to think hard about?
	11-19: Thinking about the last 6 months: Do you often lose things you need for study or work?
	11-19: Thinking about the last 6 months: Are you easily distracted?
	11-19: Thinking about the last 6 months: Are you often forgetful?
	11-19: Whether concerned at present about any aspect of concentration or activity level?
	11-19: How much have difficulties with activity or concentration upset or distressed you?
	11-19: Have difficulties with attention or activity level interfered with: How well you get along with the people you are closest to (e.g. family, partner)?
	11-19: Have difficulties with attention or activity level interfered with: Making and keeping friends?
	11-19: Have difficulties with attention or activity level interfered with: Work or study?
	11-19: Have difficulties with attention or activity level interfered with: Hobbies, sports or other leisure activities?

11-19: Have difficulties with attention or activity level made it harder for those around you (family, friends, etc.)?

Supplementary Figure 1.

Social Phobia — **youth*in*mind**

Dawba ID: 193082 — DSM-IV prediction: ++

Details: 15 years old, Male — ICD-10 prediction: ++

	Parent	Self
Any concerns?	Yes	Yes
Anxious about:		
Meeting new people	++	++
Meeting a lot of people	+	++
Eating in front of others	-	-
Speaking in class	+	+
Reading aloud in front of others	++	++
Writing in front of others	+	+
Separation or social anxiety?	Social	Social
Frightened with adults/kids	Adults and kids	Adults and kids
Can socialise with familiar people	++	++
Due to fear of embarrassment	++	++
Due to delay in speech, writing, reading	-	+
Age of onset	?	?
Duration in months	6+	6+
Blushes in social situations		-
Feels sick in social situations		-
Urgency in social situations		-
Upset when social fear is triggered	++	++
How often social fear is triggered	Rarely	Most weeks
Avoids relevant social situations	++	++
Avoidance interferes with daily life	+	++
Child thinks fear is excessive	++	+
Child upset to have social fears	++	++
Burden	+	+

>> Open-ended comments: Parent

Description of the problem
Having to speak to new people.

How often?
About once a week.

How severe?
To the point where he will actively avoid people.

Interfering with quality of life?
It's making him reluctant to leave the house.

Social Phobia - 1 — Created on: 12/07/2016 10:43

Social Phobia — **youth*in*mind**

Dawba ID: 193082 — DSM-IV prediction: ++

Details: 15 years old, Male — ICD-10 prediction: ++

Done anything about it?
Encouraged him to start doing some kind of activity.

>> Open-ended comments: Self

Description of the problem
Having to speak to people my age outside of my close friend group.

How often?
About once a day.

How severe?
To the point where I have to actively avoid social interaction by walking around people.

Interfering with quality of life?
It makes me hesitant to leave the house.

Done anything about it?
I've found a community in the online image board 4chan, specifically /r9k/ which has helped knowing that other people are in a similar situation.

Social Phobia - 2 — Created on: 12/07/2016 10:43

A fictional example of a participant DAWBA module used to showcase information collected during the interview as seen by the clinical rater.

Supplementary Figure 2.

Overview of Development and Well-Being Assessment **youth*in*mind**

Dawba ID: 193082

Details: 15 years old, Male

INFORMANTS

ID	Informant	Date	Age	Gender	About
193082	Parent	12/07/2016	15	Male	Tim
193082	Self	12/07/2016	15	Male	

SYMPTOMS, IMPACT AND DIAGNOSTIC PREDICTIONS

Predicted probability of disorder: high

	Parent		Self		Prediction	
	Symp	Imp	Symp	Imp	DSM	ICD
Autism Spectrum Disorder	++	+++			++	++
Separation Anxiety	-		-		- -	- -
Specific Phobia	+		+	-	-	-
Social Phobia	++	++	++	+++	++	++
Panic	-		++	+++	-	-
Agoraphobia	-		-		-/+	-/+
PTSD	-		-		- -	- -
OCD	-		+		-	-
BDD						
Generalised Anxiety	-		-		- -	- -
Depression	++	-	+++	+++	++	++
Deliberate Self Harm	-		-			
Bipolar						
Emotions at school						
Attachment difficulties						
Hyperactivity	-				- -	- -
Oppositional	-				-/+	-/+
Conduct	-		+		- -	- -
Anorexia / Bulimia	-		-		- -	- -
Tics	-		-		- -	- -
Psychosis						
Feeding difficulties						
Sleeping difficulties						
Elimination difficulties						
Other concerns	-		-			

CLINICAL DIAGNOSES ASSIGNED BY RATER

DSM-IV diagnoses

Asperger

Rated by: , 12/07/2016

ICD-10 diagnoses

Overview of Development and Well-Being Assessment - 1 Created on: 12/07/2016 10:42

A fictional example of a participant DAWBA used to showcase information provided by the clinical rater to allocate mental health conditions after reviewing each DAWBA module.

Hypotheses and analysis plan

Choice of the smallest effect size of interest

We chose to identify our smallest effect sizes of interest (SESOI) at different levels of explanation (i.e., theoretically, practically and clinically meaningful). Given the aim of our study to test differences in social media use across adolescents with and without mental health conditions, we believe that no single SESOI provides a comprehensive answer as to what is a meaningful effect. Our research questions hold significance for a diverse range of stakeholders, including researchers, policymakers, the general public and clinicians, each requiring insights from a different level of explanation. For instance, with a theoretically meaningful SEOSI as the benchmark, our findings can be contextualised in relation to the broader field of mental health research. On the contrary, with practically and clinically meaningful SESOIs, our results can aid decision-making for policy and clinical practice.

Therefore, while a theoretically meaningful SESOI based on everyday behaviours and their link to mental health will serve as our primary SESOI for the power calculations and equivalence tests (to allow for a clear confirmatory approach), secondary SESOIs will play an important role in supporting the interpretation of our findings.Overall, this will ensure the applicability of our study to both academic and practical domains. Of note, in the Stage 1 registered report, we reported effect sizes as Cohen's *d* while in the Stage 2 effect sizes are reported as Hedge's *g*, which is the effect size used in the TOSTER package (the formulas used to calculate both can be found at: https://aaroncaldwell.us/TOSTERpkg/articles/SMD_calcs.html).

Primary SESOI

A theoretically meaningful SESOI serves as a way to assess the significance of a specific effect size in the context of a research question, using a theoretical framework as the reference point. After an extensive scoping exercise to identify a suitable theoretical foundation, we determined that the most relevant benchmarks for our research questions are everyday behaviours linked to mental health, such as sleep and physical activity. These behaviours, much like social media use, are a regular part of daily routines but, unlike the latter, they are well-established markers of psychopathology based on both theory [4–6] and matched empirical evidence [7,8]. Consequently, if the effect size of social media use is comparable to that observed for behaviours like sleep and physical activity, we can conclude that social media use also represents an everyday behaviour that exhibits meaningful group-level differences between adolescents with and without mental health conditions.

An alternative possibility that we considered to identify the theoretical SESOI is the use of social media literature. However, despite the small effect sizes that have made headlines and attracted public attention in this space[9,10], there is still a lack of theoretical foundation as to what effects can be considered meaningful[11,12]. This combined with a lack of studies that a) directly compare social media use in clinical vs non-clinical populations (see[13]), and b) use

comparable measures of engagement to the ones examined in our study, makes it currently difficult to adequately identify the theoretical SESOI of interest solely based on social media research. We, therefore, decided to draw from research on everyday behaviours other than social media use, as expanded on below.

In the case of sleep, research[8] suggests a meta-analytic effect size of *WMD* = .48 (i.e., weighted mean difference), corresponding to *d* = 0.413 for the difference between clinical and non-clinical populations in sleep duration.[7] In terms of physical exercise, a large (*N* = 78,886) nationally representative study[14] suggests that, when compared to healthy individuals, individuals with mental health conditions are less likely to engage in recommended levels of physical activity, with a *prevalence ratio* = 0.84 after controlling for other health factors, corresponding to a medium effect size. Based on this evidence, we define *d* = 0.4 as the smallest theoretically meaningful effect size that would indicate a difference in social media use between adolescents with and without mental health conditions at least as large as that found in well-defined behavioural markers of psychopathology. We use this as our primary SESOI, informing both the power calculations and equivalence tests.

Secondary SESOIs

Given the relevance of our research question for different stakeholders (e.g., researchers, the public/policy sector, and clinicians), we also chose to identify SESOIs based on practical and clinical meaningfulness.

Practically meaningful. A practically meaningful SESOI represents the level of change in a phenomenon that individuals are likely to subjectively notice. Setting a benchmark for practical significance helps researchers define thresholds for meaningful differences or effects that are relevant in real-world scenarios. Reviews of the literature suggest that, across various physical health conditions including pain, cardiovascular and respiratory disease, when patients are asked to subjectively identify a minimal change in symptomatology, their estimates consistently fall close to half a standard deviation, corresponding to d = 0.5.[15] This value approximates the range of human discrimination first identified by Miller[16] who characterised the limit of people's abilities across a wide range of discrimination tasks as being equivalent to *d* = 0.36-0.63. Based on these grounds, *d* = 0.5 has been employed as a practically meaningful anchor of change in research on experimental design for treatment efficacy[17], media effects[18] (*d* = .41) and screen time[19]. Therefore, we set *d* = 0.5 as the benchmark for the smallest practically meaningful effect size (i.e., a difference in social media use that an individual might subjectively notice).

Clinically meaningful. An effect is clinically meaningful to the extent that it can distinguish between clinical and non-clinical populations. For example, psychotherapy research suggests that clinically meaningful interventions are those that reduce psychological distress such that an individual with a mental health diagnosis - and therefore high levels of distress - will report non-clinical levels of distress following treatment [20]. To quantify the magnitude of change that is clinically meaningful, previous research has examined Health-Related Quality of Life (HRQoL) as a transdiagnostic index of distress across clinical and non-clinical populations [21]. The HRQol is a multi-dimensional measure that captures both the physical and mental health status of individuals as well as the

overall impact that health status has on their quality of life. Studies consistently show that HRQoL differs between patients with and without mental health conditions. For instance, one meta-analysis found that patients with generalised anxiety disorder differed from controls by *d* = 1.35 in HRQoL[22]. Similarly, studies of children and adolescents diagnosed with ADHD have found differences in HRQoL of *d* = .72 and *d* = 1.05 compared to controls[23,24]. Given that reasonable SESOIs based on clinical significance are all larger than our established theoretical SESOI (*d* = 0.4), with a range of *d* = 0.72-1.35, our study will be powered to detect clinically meaningful differences in social media use across adolescents with and without mental health conditions.

Supplementary Design Table

Design Table summarising the study's research questions, hypotheses, power calculations, analyses, and conditions for interpretation.

Question	Hypothesis	Power analysis	Analysis plan	Interpretation given to different outcomes
**1) Investigate whether adolescents with any mental health condition use social media differently than those without a condition.**	Hypothesis 1.1) Adolescents with any mental health diagnosis will score higher than adolescents without a diagnosis in a) time spent on social media, b) online social comparison, c) lack of control over time spent online, d) monitoring of online feedback and e) feeling impacted by online feedback.	We calculated power given a theoretical SESOI of d = 0.4, and a sample size of at least N≥577 individuals with a diagnosis and N≥3,277 individuals without a diagnosis. Results indicated 100% power to reject effects larger than the SESOI using the *'power_t_TOST'* function for equivalence testing and 100% power to detect the SESOI in the hypothesised direction using the *'pwr.t2n.test'* function in R.	Five linear regression models will test the association between social media use (time- and engagement-based dimensions; a-e) with mental health diagnosis (two levels: *no diagnosis, diagnosis*), with the *no diagnosis* group set as the reference level.<br>We will employ equivalence tests to assess whether the 90% confidence interval (CI) for the association between social media use and mental health diagnosis lies between the equivalence bounds, -0.4 and 0.4.<br>We will employ regression models to assess whether the association between social media use and mental health diagnosis follows the hypothesised direction.	• If the CI for this association is within the equivalence bounds, it will suggest that adolescents with any mental health diagnosis do not have a meaningful difference in their social media use compared to adolescents without a diagnosis. On the contrary, if the CIs do not fall within the equivalence bounds, it will suggest that the association is of meaningful magnitude.<br>• A positive and statistically significant association between social media use and mental health diagnosis will suggest that adolescents with any mental health diagnosis score higher on the examined dimension of social media use (a-e) compared to adolescents without a diagnosis.<br>• In summary, Hypothesis 1.1 will be supported if a) the CIs for this association do not fall within the equivalence bounds, b) the association between social media use and mental health diagnosis is statistically significant, and c) the regression coefficient reflecting this association is positive.

**1) Investigate whether adolescents with any mental health condition use social media differently than those without a condition.**	Hypothesis 1.2) Adolescents with any mental health diagnosis will score lower than adolescents without a diagnosis in f) happiness about the number of online friendships, g) honest online self-disclosure, and h) authentic self-presentation online.	We calculated power given a theoretical SESOI of d = 0.4, and a sample size of at least N≥577 individuals with a diagnosis and N≥3,277 individuals without a diagnosis. Results indicated 100% power to reject effects larger than the SESOI using the *'power_t_TOST'* function for equivalence testing and 100% power to detect the SESOI in the hypothesised direction using the *'pwr.t2n.test'* function in R.	Three linear regression models will test the association between each dimension of social media engagement (f-h) and mental health diagnosis (two levels: *no diagnosis, diagnosis*), with the *no diagnosis* group set as the reference level.<br>We will employ equivalence tests to assess whether the 90% confidence interval (CI) for the association between social media use and mental health diagnosis lies between the equivalence bounds, -0.4 and 0.4.<br>We will employ regression models to assess whether the association between social media use and mental health diagnosis follows the hypothesised direction.	• If the CI for this association is within the equivalence bounds, it will suggest that adolescents with any mental health diagnosis do not have a meaningful difference in their social media use compared to adolescents without a diagnosis. On the contrary, if the CIs do not fall within the equivalence bounds, it will suggest that the association is of meaningful magnitude.<br>• A negative and statistically significant association between social media engagement and mental health diagnosis will suggest that adolescents with any mental health diagnosis score lower on the examined dimension of social media engagement (f-h) compared to adolescents without a diagnosis.<br>• In summary, Hypothesis 1.2 will be supported if a) the CIs for this association do not fall within the equivalence bounds, b) the association between social media use and mental health diagnosis is statistically significant, and c) the regression coefficient reflecting this association is negative.

**2) Investigate whether adolescents with an internalising or externalising condition use social media differently than those without a condition.**	Hypothesis 2.0) Adolescents with internalising mental health diagnoses will not differ from adolescents without a diagnosis in c) lack of control over time spent online.	We calculated power given a theoretical SESOI of d = 0.4, and a sample size of at least N≥370 individuals with internalising only diagnoses and *N*≥3,277 with no diagnosis. Results indicated 100% power to reject effects larger than the SESOI using the *'power_t_TOST'* function for equivalence testing and 100% power to detect the SESOI in the hypothesised direction using the *'pwr.t2n.test'* function in R.	One linear regression model will test the association between social media engagement (c) and diagnostic category (three levels: *no diagnosis, internalising, externalising*), with the *no diagnosis* group set as the reference level. For this hypothesis, we will examine the regression coefficient for *externalising* vs *no diagnosis*. We will employ an equivalence test to assess whether the 90% confidence interval (CI) for the association between social media use and internalising diagnosis lies between the equivalence bounds, -0.4 and 0.4.	• If the CI for this association is within the equivalence bounds, it will suggest that adolescents with an internalising diagnosis do not have a meaningful difference in their social media use compared to adolescents without a diagnosis. On the contrary, if the CIs do not fall within the equivalence bounds, it will suggest that the association is of meaningful magnitude.<br>• A non-significant association between social media use and diagnostic category (comparing *internalising* and *no diagnosis*) will suggest that adolescents with internalising diagnoses do not differ from adolescents without a diagnosis on lack of control over time spent online (c).<br>• In summary, Hypothesis 2.0 will be supported if a) the CIs for this association fall within the equivalence bounds, and b) the association between social media use and diagnostic category (comparing *internalising* and *no diagnosis*) is not statistically significant.

**2) Investigate whether adolescents with an internalising or externalising condition use social media differently than those without a condition.**	Hypothesis 2.0) Adolescents with externalising mental health diagnoses will not differ from adolescents without a diagnosis in b) online social comparison, d) monitoring of online feedback and e) feeling impacted by online feedback, g) honest online self-disclosure, and h) authentic self-presentation online.	We calculated power given a theoretical SESOI of d = 0.4, and a sample size of at least N≥199 individuals with externalising only diagnoses and *N*≥3,277 with no diagnosis. Results indicated 100% power to reject effects larger than the SESOI using the *'power_t_TOST'* function for equivalence testing and 100% power to detect the SESOI in the hypothesised direction using the *'pwr.t2n.test'* function in R.	Five linear regression models will test the association between social media use (b, d, e, g, h) and diagnostic category (three levels: *no diagnosis, internalising, externalising*), with the *no diagnosis* group set as the reference level. For this hypothesis, we will examine the regression coefficient for *externalising* vs *no diagnosis*. We will employ an equivalence test to assess whether the 90% confidence interval (CI) for the association between social media use and mental health diagnosis lies between the equivalence bounds, -0.4 and 0.4.	• If the CI for this association is within the equivalence bounds, it will suggest that adolescents with an externalising diagnosis do not have a meaningful difference in their social media use compared to adolescents without a diagnosis. On the contrary, if the CIs do not fall within the equivalence bounds, it will suggest that the association is of meaningful magnitude.<br>• A non-significant association between social media use and diagnostic category (comparing *externalising* and *no diagnosis*) will suggest that adolescents with externalising diagnoses do not differ from adolescents without a diagnosis on the considered dimension of social media engagement (b, d, e, g, h).<br>• In summary, Hypothesis 2.0 will be supported if a) the CIs for this association fall within the equivalence bounds, and b) the association between social media use and diagnostic category (comparing *externalising* and *no diagnosis*) is not statistically significant.

**2) Investigate whether adolescents with an internalising or externalising condition use social media differently than those without a condition.**	Hypothesis 2.1) Adolescents with internalising mental health diagnoses will score higher than adolescents without a diagnosis in a) time spent on social media, b) online social comparison, d) monitoring of online feedback and e) feeling impacted by online feedback.	We calculated power given a theoretical SESOI of d = 0.4, and a sample size of at least *N*≥370 individuals with internalising only diagnoses and *N*≥3,277 with no diagnosis. Results indicated 100% power to reject effects larger than the SESOI using the *'power_t_TOST'* function for equivalence testing and 100% power to detect the SESOI in the hypothesised direction using the *'pwr.t2n.test'* function in R.	Four linear regression models will test the association between social media use (time- and engagement-based dimensions; a, b, d, e) and diagnostic category (three levels: *no diagnosis, internalising, externalising*), with the *no diagnosis* group set as the reference level. For this hypothesis, we will examine the regression coefficient for *internalising* vs *no diagnosis*.<br>We will employ an equivalence test to assess whether the 90% confidence interval (CI) for the association between social media use and mental health diagnosis lies between the equivalence bounds, -0.4 and 0.4.<br>We will employ regression models to assess whether the association between social media use and diagnostic category (comparing *internalising* and *no diagnosis*) follows the hypothesised direction.	• If the CI for this association is within the equivalence bounds, it will suggest that adolescents with an internalising diagnosis do not have a meaningful difference in their social media use compared to adolescents without a diagnosis. On the contrary, if the CIs do not fall within the equivalence bounds, it will suggest that the association is of meaningful magnitude.<br>• A positive and statistically significant association between social media engagement and diagnostic category (comparing *internalising* and *no diagnosis*) will suggest that adolescents with an internalising diagnosis score higher on the examined dimension of social media engagement (a, b, d, e) compared to adolescents without a diagnosis.<br>• In summary, Hypothesis 2.1 will be supported if a) the CIs for this association do not fall within the equivalence bounds, b) the association between social media use and diagnostic category (comparing *internalising* and *no diagnosis*) is statistically significant, and c) the regression coefficient reflecting this association is positive.

**2) Investigate whether adolescents with an internalising or externalising condition use social media differently than those without a condition.**	Hypothesis 2.2) Adolescents with internalising mental health diagnoses will score lower than adolescents without a diagnosis in f) happiness about online friendships, g) honest online self-disclosure and h) authentic self-presentation online.	We calculated power given a theoretical SESOI of d = 0.4, and a sample size of at least *N*≥370 individuals with internalising only diagnosis and *N*≥3,277 with no diagnosis. Results indicated 100% power to reject effects larger than the SESOI using the *'power_t_TOST'* function for equivalence testing and 100% power to detect the SESOI in the hypothesised direction using the *'pwr.t2n.test'* function in R.	Three linear regression models will test the association between each dimension of social media engagement (f, g, h) and diagnostic category (three levels: *no diagnosis, internalising, externalising*), with the *no diagnosis* group set as the reference level. For this hypothesis, we will examine the regression coefficient for *internalising* vs *no diagnosis*.<br>We will employ an equivalence test to assess whether the 90% confidence interval (CI) for the association between social media use and mental health diagnosis lies between the equivalence bounds, -0.4 and 0.4.<br>We will employ regression models to assess whether the association between social media use and diagnostic category (comparing *internalising* and *no diagnosis*) follows the hypothesised direction.	• If the CI for this association is within the equivalence bounds, it will suggest that adolescents with an internalising diagnosis do not have a meaningful difference in their social media use compared to adolescents without a diagnosis. On the contrary, if the CIs do not fall within the equivalence bounds, it will suggest that the association is of meaningful magnitude.<br>• A negative and statistically significant association between social media engagement and diagnostic category (comparing *internalising* and *no diagnosis*) will suggest that adolescents with an internalising diagnosis score lower on the examined dimension of social media engagement (f, g, h) compared to adolescents without a diagnosis.<br>• In summary, Hypothesis 2.2 will be supported if a) the CIs for this association do not fall within the equivalence bounds, b) the association between social media use and diagnostic category (comparing *internalising* and *no diagnosis*) is statistically significant, and c) the regression coefficient reflecting this association is negative.

**2) Investigate whether adolescents with an internalising or externalising condition use social media differently than those without a condition.**	Hypothesis 2.3) Adolescents with externalising mental health diagnoses will score higher than adolescents without a diagnosis in a) time spent on social media and c) lack of control over time spent online.	We calculated power given a theoretical SESOI of d = 0.4, and a sample size of at least *N*≥199 individuals with externalising only diagnoses and *N*≥3,277 with no diagnosis. Results indicated 100% power to reject effects larger than the SESOI using the *'power_t_TOST'* function for equivalence testing and 100% power to detect the SESOI in the hypothesised direction using the *'pwr.t2n.test'* function in R.	Two linear regression models will test the association between each dimension of social media use and diagnostic category (three levels: *no diagnosis, internalising, externalising*), with the *no diagnosis* group set as the reference level. For this hypothesis, we will examine the regression coefficient for *externalising* vs *no diagnosis*.<br>We will employ an equivalence test to assess whether the 90% confidence interval (CI) for the association between social media use and mental health diagnosis lies between the equivalence bounds, -0.4 and 0.4.<br>We will employ regression models to assess whether the association between social media use and diagnostic category (comparing *externalising* and *no diagnosis*) follows the hypothesised direction.	• If the CI for this association is within the equivalence bounds, it will suggest that adolescents with externalising only diagnoses do not have a meaningful difference in their social media use compared to adolescents without a diagnosis. On the contrary, if the CIs do not fall within the equivalence bounds, it will suggest that the association is of meaningful magnitude.<br>• A positive and statistically significant association between social media engagement and diagnostic category (comparing *externalising* and *no diagnosis*) will suggest that adolescents with externalising only diagnoses score higher on the examined dimension of social media engagement (a, c) compared to adolescents without a diagnosis.<br>• In summary, Hypothesis 2.3 will be supported if a) the CIs for this association do not fall within the equivalence bounds, b) the association between social media use and diagnostic category (comparing *externalising* and *no diagnosis*) is statistically significant, and c) the regression coefficient reflecting this association is positive.

**2) Investigate whether adolescents with an internalising or externalising condition use social media differently than those without a condition.**	Hypothesis 2.4) Adolescents with externalising diagnoses will score lower than adolescents without a diagnosis in f) happiness about the number of online friendships.	We calculated power given a theoretical SESOI of d = 0.4, and a sample size of at least *N*≥199 individuals with externalising only diagnoses and *N*≥3,277 with no diagnosis. Results indicated 100% power to reject effects larger than the SESOI using the *'power_t_TOST'* function for equivalence testing and 100% power to detect the SESOI in the hypothesised direction using the *'pwr.t2n.test'* function in R.	One linear regression model will test the association between happiness about the number of online friends and diagnostic category (three levels: *no diagnosis, internalising, externalising*), with the *no diagnosis* group set as the reference level. For this hypothesis, we will examine the regression coefficient for *externalising* vs *no diagnosis*.<br>We will employ an equivalence test to assess whether the 90% confidence interval (CI) for the association between social media use and mental health diagnosis lies between the equivalence bounds, -0.4 and 0.4.<br>We will employ regression models to assess whether the association between social media use and diagnostic category (comparing *externalising* and *no diagnosis*) follows the hypothesised direction.	• If the CI for this association is within the equivalence bounds, it will suggest that adolescents with externalising only diagnoses do not have a meaningful difference in their social media use compared to adolescents without a diagnosis. On the contrary, if the CIs do not fall within the equivalence bounds, it will suggest that the association is of meaningful magnitude.<br>• A negative and statistically significant association between social media engagement and diagnostic category (comparing *externalising* and *no diagnosis*) will suggest that adolescents with externalising only diagnoses score lower on the examined dimension of social media engagement (f) compared to adolescents without a diagnosis.<br>• In summary, Hypothesis 2.4 will be supported if a) the CIs for this association do not fall within the equivalence bounds, b) the association between social media use and diagnostic category (comparing *externalising* and *no diagnosis*) is statistically significant, b) the regression coefficient reflecting this association is negative.

**3) Investigate whether adolescents with an internalising condition use social media differently than those with an externalising condition.**	Hypothesis 3.0) Adolescents with internalising diagnoses will not differ from adolescents with an externalising diagnoses in a) time spent on social media and f) happiness about the number of online friendships.	We calculated power given a theoretical SESOI of d = 0.4, and a sample size of at least *N*≥370 individuals with internalising only and *N*≥199 with externalising only diagnoses. Results indicated 96% power to reject effects larger than the SESOI using the *'power_t_TOST'* function for equivalence testing and 99.8% power to detect the SESOI in the hypothesised direction using the *'pwr.t2n.test'* function in R.	Two linear regression models will test the association between each dimension of social media engagement and diagnostic category (three levels: *no diagnosis, internalising, externalising*), with the *internalising diagnosis* group set as the reference level. For this hypothesis, we will examine the regression coefficient for *internalising* vs *externalising*. We will employ an equivalence test to assess whether the 90% confidence interval (CI) for the association between social media use and mental health diagnosis lies between the equivalence bounds, -0.4 and 0.4.	• If the CI for this association is within the equivalence bounds, it will suggest that adolescents with externalising diagnoses do not have a meaningful difference in their social media use compared to adolescents with internalising diagnoses. On the contrary, if the CIs do not fall within the equivalence bounds, it will suggest that the association is of meaningful magnitude.<br>• A non-significant association between social media use and diagnostic category (comparing *internalising* and *externalising*) will suggest that adolescents with internalising diagnoses do not differ from adolescents with externalising diagnoses on the considered dimension of social media engagement (a, f).<br>• In summary, Hypothesis 3.0 will be supported if a) the CIs for this association fall within the equivalence bounds, and b) the association between social media use and diagnostic category (comparing *internalising* and *externalising*) is not statistically significant.

**3) Investigate whether adolescents with an internalising condition use social media differently than those with an externalising condition.**	Hypothesis 3.1) Adolescents with internalising diagnoses will score higher than adolescents with externalising diagnoses in b) online social comparison, d) monitoring of online feedback, and e) feeling impacted by online feedback.	We calculated power given a theoretical SESOI of d = 0.4, and a sample size of at least *N*≥370 individuals with internalising only and *N*≥199 with externalising only diagnoses. Results indicated 96% power to reject effects larger than the SESOI using the *'power_t_TOST'* function for equivalence testing and 99.8% power to detect the SESOI in the hypothesised direction using the *'pwr.t2n.test'* function in R.	Three linear regression models will test the association between each dimension of social media engagement and diagnostic category (three levels: *no diagnosis, internalising, externalising*), with the *externalising diagnosis* group set as the reference level. For this hypothesis, we will examine the regression coefficient for *internalising* vs *externalising*. We will employ an equivalence test to assess whether the 90% confidence interval (CI) for the association between social media use and mental health diagnosis lies between the equivalence bounds, -0.4 and 0.4.<br>We will employ regression models to assess whether the association between social media use and diagnostic category (comparing *internalising* and *externalising*) follows the hypothesised direction.	• If the CI for this association is within the equivalence bounds, it will suggest that adolescents with internalising only diagnoses do not have a meaningful difference in their social media use compared to adolescents with externalising only diagnoses. On the contrary, if the CIs do not fall within the equivalence bounds, it will suggest that the association is of meaningful magnitude.<br>• A positive and statistically significant association between social media engagement and diagnostic category (comparing *internalising* and *externalising*) will suggest that adolescents with internalising only diagnoses score higher on the examined dimension of social media engagement (b, d, e) compared to adolescents with externalising only diagnoses.<br>• In summary, Hypothesis 3.1 will be supported if a) the CIs for this association do not fall within the equivalence bounds, b) the association between social media use and diagnostic category (comparing *internalising* and *externalising*) is statistically significant, and c) the regression coefficient reflecting this association is positive.

**3) Investigate whether adolescents with an internalising condition use social media differently than those with an externalising condition.**	Hypothesis 3.2) Adolescents with internalising diagnoses will score lower than adolescents with externalising diagnoses in c) lack of control over time spent online, g) online honest self-disclosure and h) authentic self-presentation online.	We calculated power given a theoretical SESOI of d = 0.4, and a sample size of at least *N*≥370 individuals with internalising only and *N*≥199 with externalising only diagnoses. Results indicated 96% power to reject effects larger than the SESOI using the *'power_t_TOST'* function for equivalence testing and 99.8% power to detect the SESOI in the hypothesised direction using the *'pwr.t2n.test'* function in R.	Three linear regression models will test the association between each dimension of social media engagement and diagnostic category (three levels: *no diagnosis, internalising, externalising*), with the *externalising diagnosis* group set as the reference level. For this hypothesis, we will examine the regression coefficient for *internalising* vs *externalising*. We will employ an equivalence test to assess whether the 90% confidence interval (CI) for the association between social media use and mental health diagnosis lies between the equivalence bounds, -0.4 and 0.4.<br>We will employ regression models to assess whether the association between social media use and diagnostic category (comparing *internalising* and *externalising*) follows the hypothesised direction.	• If the CI for this association is within the equivalence bounds, it will suggest that adolescents with internalising only diagnoses do not have a meaningful difference in their social media use compared to adolescents with externalising only diagnoses. On the contrary, if the CIs do not fall within the equivalence bounds, it will suggest that the association is of meaningful magnitude.<br>• A negative and statistically significant association between social media engagement and diagnostic category (comparing *internalising* and *externalising*) will suggest that adolescents with internalising only diagnoses lower higher on the examined dimension of social media engagement (c, g, h) compared to adolescents with externalising only diagnoses.<br>• In summary, Hypothesis 3.2 will be supported if a) the CIs for this association do not fall within the equivalence bounds, b) the association between social media use and diagnostic category (comparing *internalising* and *externalising*) is statistically significant, b) the regression coefficient reflecting this association is negative.

Exploratory analyses for specific diagnoses or diagnostic categories

The Registered Report will include exploratory analyses on specific conditions, as reported below. Of note, we decided to include these analyses as exploratory because a) our ability to conduct them will be conditioned on sample size and power, and b) we do not have specific hypotheses related to these conditions.

To increase the clinical utility of this Registered Report we will run additional exploratory analyses examining responses to all social media questions (a-g) separately for individual mental health conditions (e.g., major depressive disorder).First, we will present descriptive statistics for each specific condition, as long as there are at least 10 individuals with that condition. Next, for individual conditions with sufficient sample size, we will conduct follow up analyses to test whether individuals falling within each group respond differently to each social media question (a-g) than individuals with no condition. As comorbid mental health conditions are extremely common[25], we will include individuals with multiple conditions in these analyses.

Supplementary results (Stage 2)

Descriptive statistics

Supplementary Table 5.

Descriptive statistics (mean, standard deviation, sample size) for each social media measure by binary diagnostic category (no vs any diagnosis).

**Social Media**	**Statistic**	**No mental health condition**	**Any mental health condition**
Time spent weekly	mean	1.89	2.76
	sd	1.65	2.09
	n	2786	510
	5% CI	1.86	2.67
	95% CI	1.92	2.85
Social Comparison	mean	2.26	2.69
	sd	1.30	1.49
	n	2694	496
	5% CI	2.24	2.63
	95% CI	2.29	2.75
Lack of control over time spent	mean	3.17	3.56
	sd	1.42	1.45
	n	2732	494
	5% CI	3.15	3.49
	95% CI	3.20	3.62
Monitoring of feedback	mean	2.94	3.05
	sd	1.47	1.53
	n	2670	483
	5% CI	2.91	2.98
	95% CI	2.96	3.12
Impact of feedback on mood	mean	1.83	2.21
	sd	1.17	1.42
	n	2686	486
	5% CI	1.81	2.15
	95% CI	1.85	2.27
	mean	4.59	4.26

Happy about friends	sd	0.78	1.00
	n	2714	483
	5% CI	4.58	4.21
	95% CI	4.61	4.30
Honest self-disclosure	mean	3.64	3.25
	sd	1.22	1.39
	n	2644	485
	5% CI	3.62	3.19
	95% CI	3.67	3.31
Authentic self-presentation	mean	3.81	3.56
	sd	1.21	1.33
	n	2602	475
	5% CI	3.78	3.50
	95% CI	3.83	3.62

Supplementary Table 6.

Descriptive statistics (mean, standard deviation, sample size, 95% confidence interval for the mean) for each social media measure by categorical diagnostic category (internalising-externalising between group comorbidity, externalising only, internalising only, other diagnoses, no diagnosis).

		**Diagnostic category**				
Social Media	Statistic	Between comorbidity	Externalising	Internalising	Other conditions	No condition
Time spent weekly	mean	3.74	2.47	3.02	1.43	1.89
	sd	2.53	2.10	1.98	1.25	1.65
	n	57	102	278	73	2786
	5% CI	3.42	2.27	2.90	1.29	1.86
	95% CI	4.07	2.67	3.13	1.57	1.92
Social comparison	mean	2.71	2.13	3.03	2.10	2.26
	sd	1.50	1.25	1.50	1.36	1.30
	n	55	98	276	67	2694
	5% CI	2.51	2.01	2.94	1.94	2.24
	95% CI	2.91	2.25	3.11	2.27	2.29
Lack of control over time spent	mean	3.66	3.44	3.78	2.77	3.17
	sd	1.53	1.45	1.38	1.42	1.42
	n	53	95	276	70	2732
	5% CI	3.46	3.30	3.70	2.61	3.15
	95% CI	3.87	3.59	3.86	2.94	3.20
Monitoring of feedback	mean	3.00	3.07	3.14	2.71	2.94
	sd	1.63	1.51	1.51	1.57	1.47
	n	55	92	271	65	2670
	5% CI	2.79	2.91	3.05	2.52	2.91
	95% CI	3.21	3.22	3.22	2.90	2.96
Impact of feedback on mood	mean	2.25	2.17	2.33	1.70	1.83
	sd	1.42	1.36	1.47	1.21	1.17
	n	55	93	272	66	2686
	5% CI	2.07	2.04	2.25	1.55	1.81

	95% CI	2.44	2.31	2.42	1.84	1.85
Happy about friends	mean	4.40	4.47	4.19	4.15	4.59
	sd	1.04	0.75	1.01	1.20	0.78
	n	53	91	272	67	2714
	5% CI	4.26	4.40	4.13	4.01	4.58
	95% CI	4.54	4.55	4.25	4.29	4.61
Honest self-disclosure	mean	3.02	3.38	3.24	3.31	3.64
	sd	1.54	1.31	1.39	1.35	1.22
	n	53	94	271	67	2644
	5% CI	2.81	3.25	3.15	3.15	3.62
	95% CI	3.22	3.51	3.32	3.47	3.67
Authentic self-presentation	mean	3.75	3.70	3.56	3.25	3.81
	sd	1.40	1.15	1.36	1.38	1.21
	n	51	92	267	65	2602
	5% CI	3.55	3.58	3.48	3.08	3.78
	95% CI	3.94	3.81	3.64	3.41	3.83

Note. The main column title is reported in bold, the social media items are reported in italics, and all the titles (including subtitles) for the columns and rows are highlighted in grey for clarity. The same structure is used in the following tables.

Supplementary Table 7.

Descriptive statistics (mean, standard deviation, sample size, 95% confidence interval for the mean) for each social media item by sex (male vs female) and binary diagnostic category (any vs no diagnosis).

		**No mental health condition**		**Any mental health condition**	
Social Media	Statistic	Male	Female	Male	Female
Time spent weekly	mean	1.65	2.14	2.20	3.27
	sd	1.50	1.76	1.91	2.11
	n	1397	1389	241	269
	5% CI	1.61	2.09	2.08	3.14
	95% CI	1.68	2.19	2.32	3.39
Social comparison	mean	2.06	2.47	2.24	3.07
	sd	1.20	1.37	1.29	1.55
	n	1350	1344	229	267
	5% CI	2.03	2.43	2.16	2.98
	95% CI	2.09	2.50	2.33	3.16
Lack of control over time spent	mean	2.88	3.46	3.15	3.90
	sd	1.41	1.38	1.45	1.37
	n	1369	1363	226	268
	5% CI	2.85	3.42	3.06	3.82
	95% CI	2.92	3.50	3.24	3.98
Monitoring of feedback	mean	2.83	3.05	2.93	3.15
	sd	1.45	1.49	1.53	1.54
	n	1335	1335	222	261
	5% CI	2.79	3.01	2.83	3.06
	95% CI	2.86	3.09	3.03	3.24
Impact of feedback on mood	mean	1.81	1.84	2.09	2.31
	sd	1.16	1.18	1.38	1.45
	n	1344	1342	224	262

	5% CI	1.78	1.81	2.00	2.22
	95% CI	1.84	1.87	2.18	2.40
Happy about friends	mean	4.56	4.62	4.26	4.26
	sd	0.80	0.76	1.03	0.99
	n	1359	1355	220	263
	5% CI	4.54	4.60	4.19	4.20
	95% CI	4.58	4.64	4.33	4.32
Honest self-disclosure	mean	3.67	3.61	3.32	3.20
	sd	1.22	1.22	1.40	1.37
	n	1333	1311	224	261
	5% CI	3.64	3.58	3.23	3.11
	95% CI	3.71	3.65	3.41	3.28
Authentic self-presentation	mean	3.65	3.96	3.46	3.65
	sd	1.26	1.14	1.32	1.34
	n	1303	1299	220	255
	5% CI	3.62	3.93	3.37	3.57
	95% CI	3.68	3.99	3.54	3.73

Supplementary Table 8.

Descriptive statistics (mean, standard deviation, sample size, 95%CI) for each social media item by sex (male vs female) and categorical diagnostic category (internalising-externalising between group comorbidity, externalising only, internalising only, other diagnoses, no diagnosis).

		**Between Comorbidity**		**Externalising**		**Internalising**		**Other**		**No condition**	
Social Media	Statistic	Male	Female	Male	Female	Male	Female	Male	Female	Male	Female
Time spent weekly	mean	2.82	4.69	2.20	3.16	2.56	3.20	1.25	1.48	1.65	2.14
	sd	2.36	2.39	2.01	2.21	1.87	2.00	1.35	1.17	1.50	1.76
	n	29	28	73	29	80	198	59	14	1397	1389
	5% CI	2.39	4.25	1.98	2.75	2.36	3.06	1.23	1.30	1.61	2.09
	95% CI	3.25	5.14	2.43	3.56	2.76	3.34	1.55	1.90	1.68	2.19
Social comparison	mean	2.50	2.93	2.13	2.08	2.38	3.28	2.08	2.21	2.06	2.47
	sd	1.40	1.59	1.19	1.34	1.30	1.51	1.34	1.48	1.2	1.37
	n	28	27	71	53	77	199	53	14	1350	1344
	5% CI	2.24	2.62	1.99	1.88	2.23	3.17	1.90	1.81	2.03	2.43
	95% CI	2.76	3.23	2.26	2.25	2.52	3.38	2.25	2.61	2.09	2.5
Lack of control over time spent	mean	3.12	4.19	3.28	2.68	3.40	3.92	2.68	3.15	2.88	3.46
	sd	1.53	1.36	1.42	1.44	1.40	1.35	1.44	1.28	1.41	1.38
	n	26	27	68	57	75	201	57	13	1369	1363
	5% CI	2.82	3.93	3.11	3.57	3.24	3.82	2.50	2.79	2.85	3.42
	95% CI	3.41	4.44	3.45	2.87	3.56	4.01	2.87	3.51	2.92	3.5
Monitoring of feedback	mean	2.96	3.04	3.05	2.70	2.97	3.20	2.7	2.75	2.83	3.05
	sd	1.62	1.68	1.49	1.58	1.50	1.51	1.58	1.6	1.45	1.49
	n	28	27	66	53	75	196	53	12	1335	1335
	5% CI	2.66	2.72	2.87	2.81	2.81	3.09	2.49	2.28	2.79	3.01
	95% CI	3.27	3.36	3.22	2.91	3.14	3.30	2.91	3.22	2.86	3.09
Impact of feedback on mood	mean	2.00	2.52	2.29	1.69	2.23	2.37	1.69	1.75	1.81	1.84
	sd	1.36	1.45	1.38	1.24	1.43	1.48	1.24	1.14	1.16	1.18
	n	28	27	68	54	74	198	54	12	1344	1342

	5% CI	1.75	2.24	2.13	1.59	2.07	2.27	1.52	1.41	1.78	1.81
	95% CI	2.25	2.79	2.46	2.09	2.39	2.48	1.85	2.09	1.84	1.87
Happy about friends	mean	4.11	4.69	4.42	4.59	4.31	4.14	4.07	4.46	4.56	4.62
	sd	1.25	0.68	0.79	0.64	0.88	1.05	1.29	0.66	0.8	0.76
	n	27	26	64	27	75	197	54	13	1359	1355
	5% CI	3.87	4.56	4.33	4.47	4.21	4.07	3.90	4.28	4.54	4.60
	95% CI	4.35	4. 82	4.52	4.71	4.41	4.21	4.24	4.65	4.58	4.64
Honest self-disclosure	mean	2.78	3.27	3.42	3.30	3.51	3.13	3.19	3.85	3.67	3.61
	sd	1.53	1.54	1.29	1.38	1.43	1.37	1.39	1.07	1.22	1.22
	n	27	26	67	27	76	195	54	13	1333	1311
	5% CI	2.49	2.97	3.26	3.03	3.35	3.03	3.00	3.55	3.64	3.58
	95% CI	3.07	3.57	3.57	3.56	3.67	3.22	3.37	4.15	3.71	3.65
Authentic self-presentation	mean	3.58	3.89	3.60	3.96	3.50	3.58	3.15	3.62	3.65	3.96
	sd	1.50	1.31	1.15	1.12	1.34	1.37	1.41	1.26	1.26	1.14
	n	24	27	68	24	76	191	52	13	1303	1299
	5% CI	3.28	3.64	3.47	3.73	3.35	3.49	2.96	3.26	3.62	3.93
	95% CI	3.89	4.14	3.74	4.19	3.65	3.68	3.34	3.97	3.68	3.99

Violin Plots

Supplementary Figure 3

Violin plots for social media use in adolescents without (No) and with (Yes) any mental health condition.

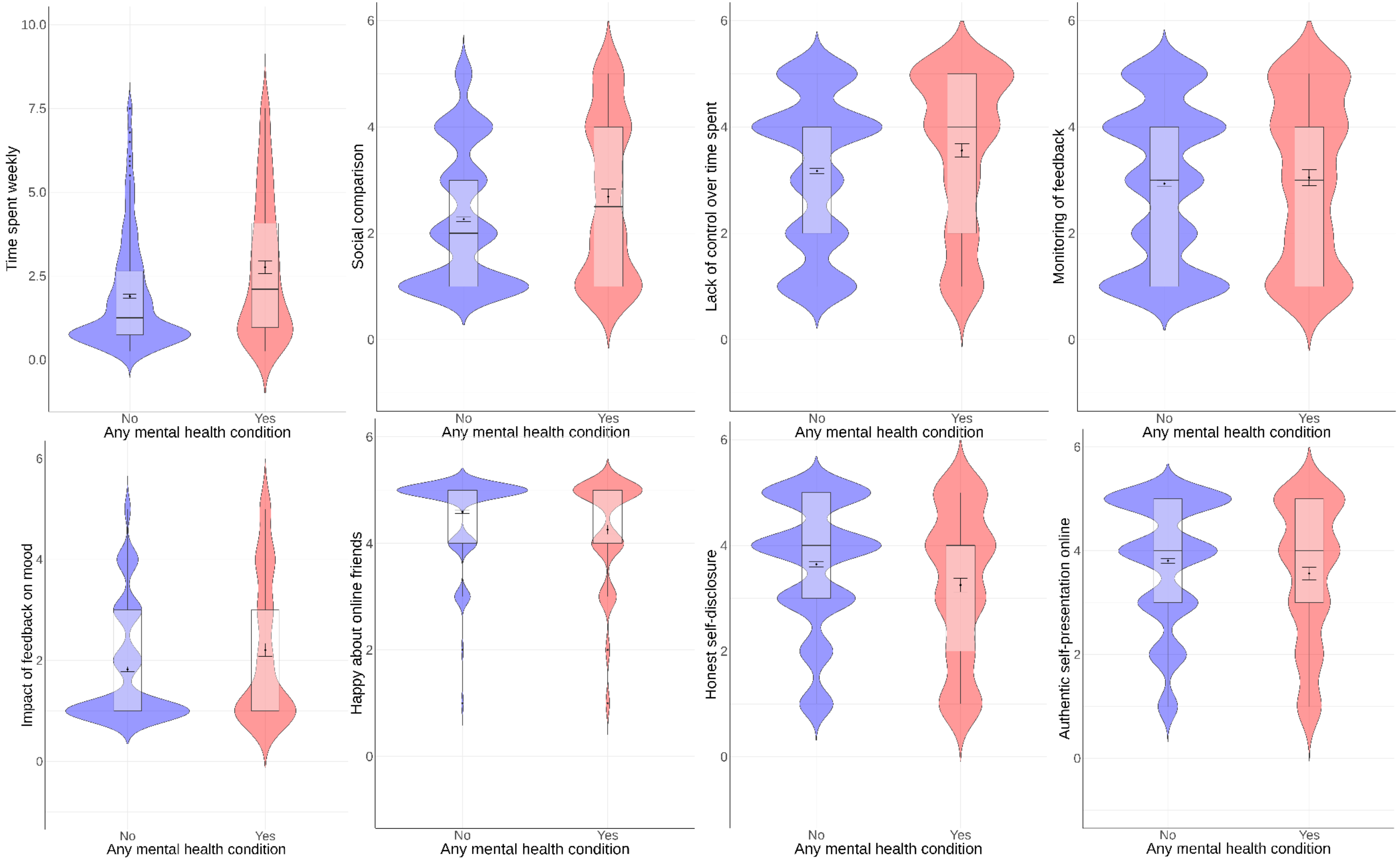

Supplementary Figure 4

Violin plots for social media use by mental health condition (Internalising, Externalising, None)

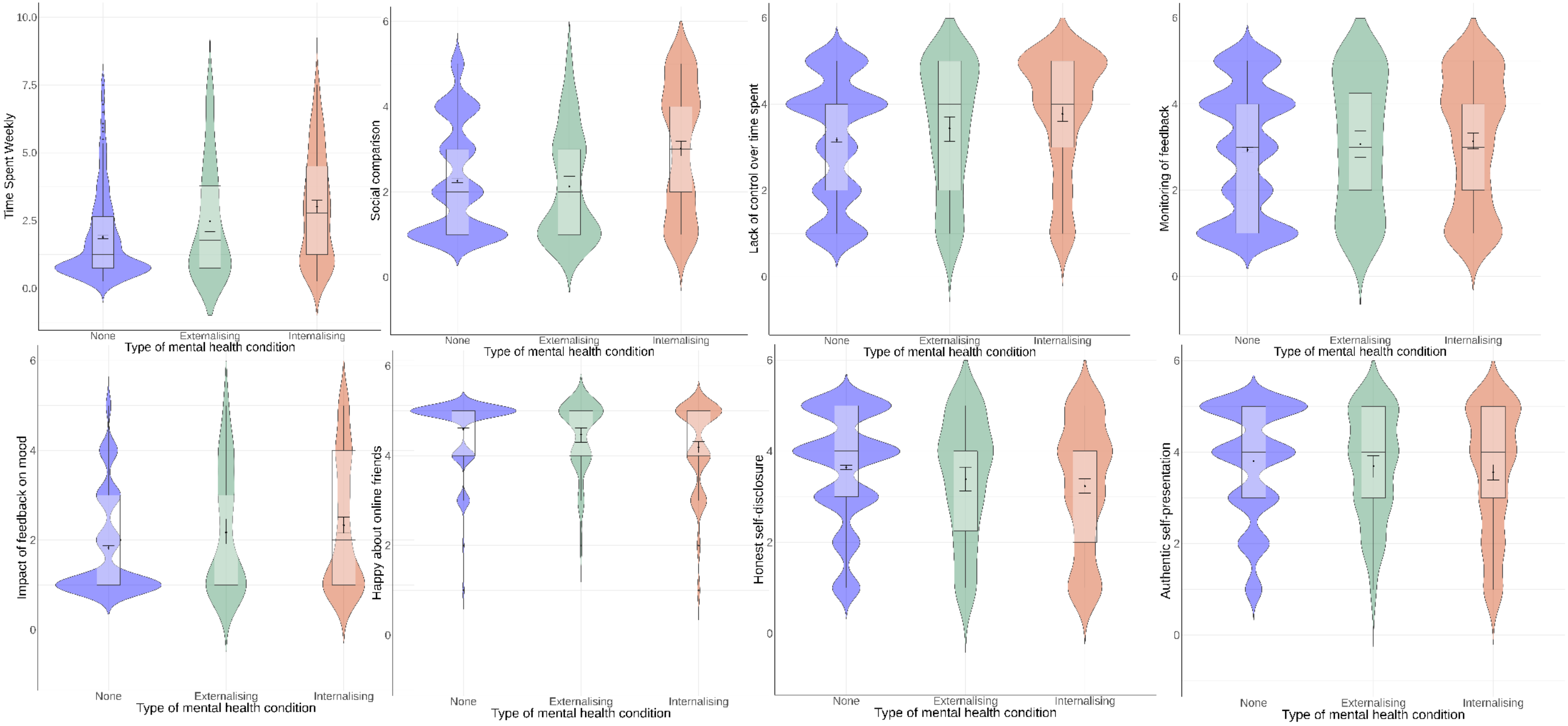

Supplementary Figure 5

Violin plots for social media use in adolescents with (Yes) and without (No) mental health conditions split by sex (male/female).

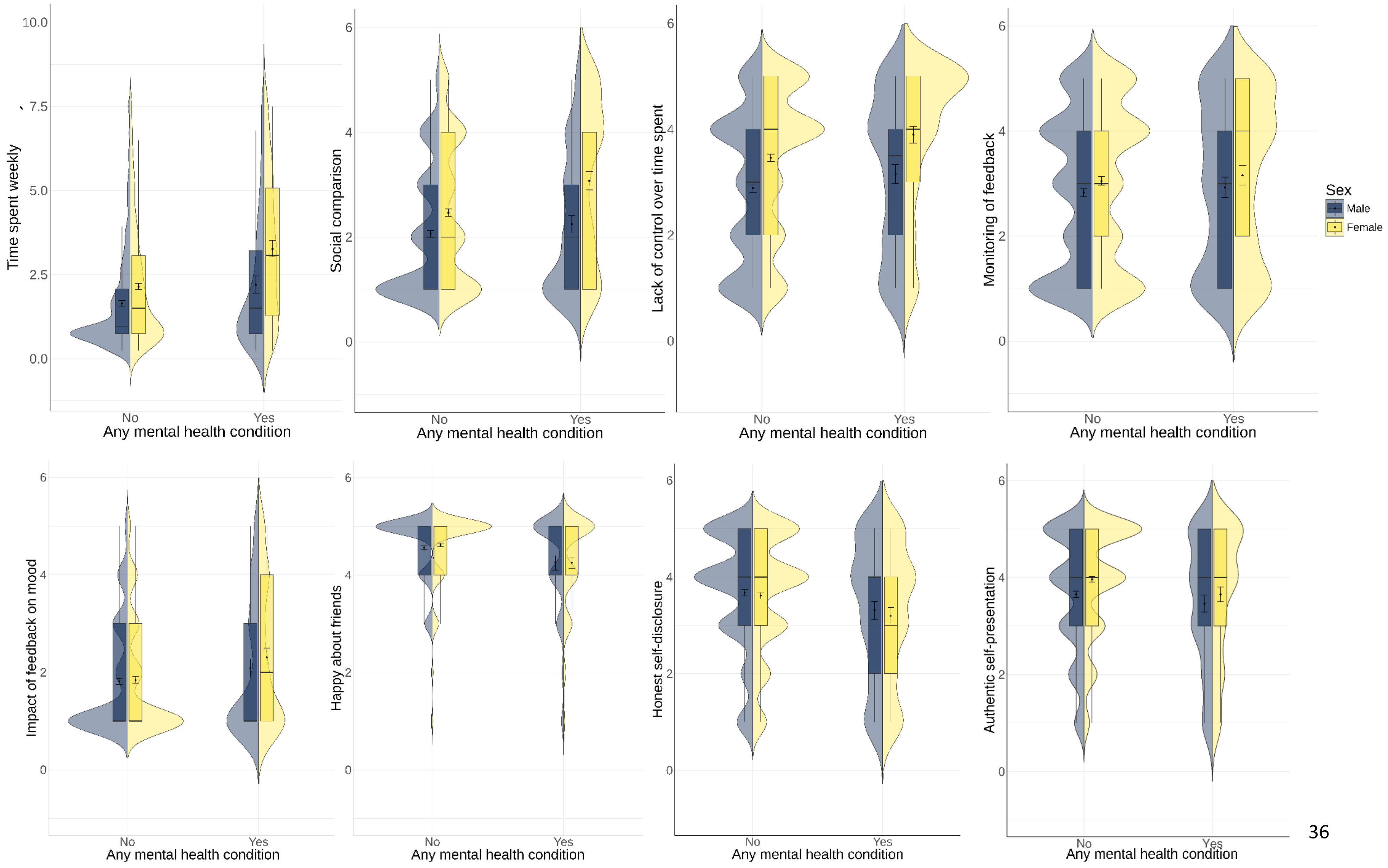

Note. Violin plots for the dimensions of social media use. Supplementary Figure 3 displays plots for adolescents with vs without a mental health condition, Supplementary Figure 4 displays plots for adolescents with internalising, externalising vs no mental health condition, and Supplementary Figure 5 displays plots for adolescents with any vs no mental health condition split by sex (Male vs Female). In each violin plot, the x-axis represents the grouping variable (Any Mental Health Condition: Yes/No, or Type of Mental Health Condition: None, Externalising, Internalising). The y-axis displays each social media dimension, measured on a 5-point Likert scale, except time spent weekly, which is measured on a 9-point Likert scale. The plot presents violin shapes to show the distribution of data, with overlaid boxplots indicating the median and interquartile range, and black dots representing the mean with bootstrapped 95% confidence intervals. The exact sample size for each subcategory is reported in Supplementary Tables 5-8 and varies for each considered dimension of social media use.

Supplementary Table 9.

Overview of primary hypotheses and confirmatory test results.

	**H1: Any vs no condition**		**H2: Internalising vs no condition**		**H2: Externalising vs no condition**		**H3: Internalising vs externalising condition**	
Social Media	Hypothesis	Result	Hypothesis	Result	Hypothesis	Result	Hypothesis	Result
Time spent weekly	*Any condition higher*	Confirmed	*Internalising higher*	Confirmed	*Externalising higher*	Confirmed	*No differences*	Confirmed (No differences)
Social Comparison	*Any condition higher*	Not confirmed (No differences)	*Internalising higher*	Confirmed	*No differences*	Confirmed	*Internalising higher*	Confirmed
Lack of control over time spent	*Any condition higher*	Not confirmed (No differences)	*No differences*	Not confirmed (Internalising higher)	*Externalising higher*	Not confirmed (No differences)	*Internalising lower*	Not confirmed (No differences)
Monitoring of feedback	*Any condition higher*	Not confirmed (No differences)	*Internalising higher*	Not confirmed (No differences)	*No differences*	Confirmed	*Internalising higher*	Not confirmed (No differences)
Impact of feedback on mood	*Any condition higher*	Not confirmed (No differences)	*Internalising higher*	Confirmed	*No differences*	Not confirmed (Externalising higher)	*Internalising higher*	Not confirmed (No differences)
Happy about friends	*Any condition lower*	Confirmed	*Internalising lower*	Confirmed	*Externalising lower*	Not confirmed (No differences)	*No differences*	Not confirmed (Internalising lower)
Honest self-disclosure	*Any condition lower*	Not confirmed (No differences)	*Internalising lower*	Confirmed	*No differences*	Confirmed	*Internalising lower*	Not confirmed (No differences)
Authentic self-presentation	*Any condition lower*	Not confirmed (No differences)	*Internalising lower*	Not confirmed (No differences)	*No differences*	Confirmed	*Internalising lower*	Not confirmed (No differences)

Note. The hypotheses pre-registered in the Stage 1 Registered Report are reported in italics.

Non-parametric tests

Supplementary Table 10.

Assumptions tests for the pre-registered regression models.

		**Independence of residual erros**		**Heteroscedasticity of residuals**		**Normality of residuals**	
Social Media	Hypothesis	Durbin Watson (test statistic)	Durbin Watson (p value)	Breusch-Pagan test (statistic)	Breusch-Pagan test (p value)	Shapiro Wilk (test statistic)	Shapiro Wilk (p value)
**H1: Adolescents with vs without mental health conditions**							
Time spent weekly	H1a	1.93	0.05	64.47	< .0001	0.863	< .0001
Social comparison	H1b	1.93	0.036	18.563	< .0001	0.873	< .0001
Lack of control over time spent	H1c	1.93	0.066	0.416	0.519	0.888	< .0001
Monitoring of feedback	H1d	1.93	0.076	1.303	0.254	0.873	< .0001
Impact of feedback on mood	H1e	1.93	0.036	41.121	< .0001	0.783	< .0001
Happy about friends	H1f	2.00	0.928	71.145	< .0001	0.679	< .0001
Honest self-disclosure	H1g	1.93	0.056	15.307	< .0001	0.897	< .0001
Authentic self-presentation	H1h	1.90	0.014	8.555	0.003	0.871	< .0001
**H2: Adolescents with internalising/externalising vs no condition**							
Time spent weekly	H2a	1.91	0.006	30.051	< .0001	0.861	< .0001

Social comparison	H2b	1.92	0.026	13.719	0.00021	0.868	< .0001
Lack of control over time spent	H2c	1.94	0.096	0.316	0.57408	0.887	< .0001
Monitoring of feedback	H2d	1.93	0.054	0.301	0.58353	0.877	< .0001
Impact of feedback on mood	H2e	1.92	0.028	39.585	< .0001	0.782	< .0001
Happy about friends	H2f	2.00	0.900	46.321	< .0001	0.666	< .0001
Honest self-disclosure	H2g	1.92	0.034	10.311	0.00132	0.89	< .0001
Authentic self-presentation	H2h	1.92	0.016	6.676	0.00977	0.865	< .0001
**H3: Adolescents with internalising vs externalising condition**							
Time spent weekly	H3a	1.91	0.016	30.051	< .0001	0.861	< .0001
Social comparison	H3b	1.92	0.022	13.719	0.0002	0.868	< .0001
Lack of control over time spent	H3c	1.94	0.084	0.316	0.574	0.887	< .0001
Monitoring of feedback	H3d	1.93	0.074	0.301	0.584	0.877	< .0001
Impact of feedback on mood	H3e	1.92	0.036	39.585	< .0001	0.782	< .0001
Happy about friends	H3f	2.00	0.932	46.321	< .0001	0.666	< .0001
Honest self-disclosure	H3g	1.92	0.042	10.311	0.001	0.890	< .0001
Authentic self-presentation	H3h	1.92	0.022	6.676	0.010	0.865	< .0001

Supplementary Table 11.

Non-parametric tests (Brunner-Munzel tests) applied to null hypothesis significance testing and equivalence testing for our primary hypotheses.

**H1: Adolescents with vs without mental health conditions**										
	**Brunner-Munzel NHST**					**Brunner-Munzel equivalence testing**		**Effect size (SESOIs; mu = 0.4-0.6)**		
Social Media	t value	df	b	std.Error	p	t	p	mu	mu (90%CI lower)	mu (90%CI upper)
Time spent weekly	8.82	654.83	0.62	0.805	3.62E-25	1.70	0.955	0.62	0.60	0.65
Social comparison	5.45	625.83	0.58	0.806	3.54E-08	-1.55	0.060	0.58	0.55	0.60
Lack of control over time spent	5.87	633.13	0.58	0.806	3.46E-09	-1.17	0.121	0.58	0.56	0.61
Monitoring of feedback	1.62	634.80	0.52	0.807	0.054	-5.34	6.53E-08	0.52	0.5	0.55
Impact of feedback on mood	4.93	611.25	0.57	0.767	5.22E-07	-2.41	0.008	0.57	0.54	0.59
Happy about friends	-7.70	618.42	0.40	0.705	2.801E-14	0.33	0.371	0.40	0.38	0.42
Honest self-disclosure	-5.46	630.27	0.42	0.795	3.431E-08	1.57	0.058	0.42	0.40	0.45
Authentic self-presentation	-3.42	630.15	0.45	0.787	3.000E-04	3.63	0.000	0.45	0.43	0.47
**H2: Adolescents with internalising vs no condition**										
	**Brunner-Munzel NHST**					**Brunner-Munzel equivalence testing**		**Effect size (SESOIs; mu = 0.4-0.6)**		
Social Media	t value	df	b	std.Error	p	t	p	mu	mu (90%CI lower)	mu (90%CI upper)

Time spent weekly	10.29	326.026	0.68	0.95	0.01E-25	4.48	0.999	0.68	0.65	0.71
Social comparison	7.98	313.76	0.65	0.99	1.43E-14	2.46	0.993	0.64	0.61	0.67
Lack of control over time spent	7.18	320.14	0.63	0.98	4.84E-12	1.58	0.942	0.63	0.60	0.66
Monitoring of feedback	2.15	319.91	0.54	1.00	0.016	-3.29	0.000	0.54	0.51	0.57
Impact of feedback on mood	5.14	307.97	0.59	0.97	2.42E-07	-0.44	0.329	0.59	0.56	0.62
Happy about friends	-7.20	311.55	0.38	0.89	2.31E-12	-1.09	0.861	0.38	0.36	0.41
Honest self-disclosure	-4.39	315.60	0.42	0.99	7.74E-06	1.05	0.148	0.42	0.39	0.45
Authentic self-presentation	-2.49	311.06	0.45	1.00	0.007	2.88	0.000	0.45	0.42	0.48
**H2: Adolescents with externalising vs no condition**										
	**Brunner-Munzel NHST**					**Brunner-Munzel equivalence testing**		**Effect size (SESOIs; mu = 0.4-0.6)**		
Social Media	t value	df	b	std.Error	p	t	p	mu	mu (90%CI lower)	mu (90%CI upper)
Time spent weekly	1.96	105.71	0.56	1.73	0.026	-1.15	0.126	0.56	0.51	0.62
Social comparison	-1.07	104.382	0.47	1.48	0.288	2.50	0.007	0.47	0.42	0.52
Lack of control over time spent	1.86	99.311	0.56	1.64	0.033	-1.39	0.083	0.56	0.51	0.61
Monitoring of feedback	0.86	96.713	0.53	1.62	0.392	-2.39	0.009	0.53	0.48	0.58

Impact of feedback on mood	2.18	96.668	0.56	1.55	0.031	-1.21	0.115	0.56	0.52	0.61
Happy about friends	-2.23	96.028	0.44	1.36	0.014	1.68	0.048	0.44	0.40	0.49
Honest self-disclosure	-1.86	99.064	0.44	1.56	0.065	1.49	0.070	0.44	0.39	0.49
Authentic self-presentation	-1.23	98.529	0.47	1.48	0.224	2.28	0.012	0.47	0.42	0.51
**H3: Internalising vs externalising condition**										
	**Brunner-Munzel NHST**					**Brunner-Munzel equivalence testing**		**Effect size**		
Social Media	t value	df	b	std.Error	p	t	p	mu	mu (90%CI lower)	mu (90%CI upper)
Time spent weekly	2.80	155.84	0.59	0.67	0.008	-0.11	0.457	0.60	0.54	0.65
Social comparison	5.95	213.84	0.67	0.56	5.487E-09	2.49	0.993	0.67	0.62	0.72
Lack of control over time spent	2.05	160.23	0.57	0.64	0.979	-0.98	0.164	0.57	0.51	0.62
Monitoring of feedback	0.35	157.12	0.51	0.65	0.362	-2.57	0.006	0.51	0.46	0.57
Impact of feedback on mood	0.95	171.15	0.53	0.61	0.171	-2.18	0.015	0.53	0.48	0.58
Happy about friends	-2.33	185.75	0.43	0.56	0.021	1.05	0.148	0.43	0.38	0.48
Honest self-disclosure	-0.82	172.80	0.47	0.63	0.206	2.20	0.015	0.47	0.42	0.53
Authentic self-presentation	-0.48	191.95	0.48	0.61	0.314	2.61	0.005	0.48	0.43	0.54

Supplementary Table 12.

Summary of discrepancies between pre-registered parametric tests and non-parametric test results for Question 1.

	**H1: Adolescents with vs without mental health conditions**		
Social Media	Hypothesis	Result parametric	Result not parametric
Time spent weekly	*Clinical higher*	Confirmed	Confirmed
Social comparison	*Clinical higher*	Not confirmed	Confirmed* (but marginally; p = 0.06 for EQV indicating close to no differences)
Lack of control over time spent	*Clinical higher*	Not confirmed	Confirmed*
Monitoring of feedback	*Clinical higher*	Not confirmed	Not confirmed
Impact of feedback on mood	*Clinical higher*	Not confirmed	Not confirmed
Happy about friends	*Clinical lower*	Confirmed	Confirmed
Honest self-disclosure	*Clinical lower*	Not confirmed	Confirmed*
Authentic self-presentation	*Clinical lower*	Not confirmed	Not confirmed

Note.* denotes tests where we found a discrepancy. The hypotheses preregistered in the Stage 1 protocol are reported in italics.

Supplementary Table 13.
Summary of discrepancies between pre-registered parametric tests and non-parametric test results for Question 2.

	**H2: Adolescents with internalising vs no condition**			**H2: Adolescents with externalising vs no condition**		
Social Media	Hypothesis	Result	Result not parametric	Hypothesis	Result	Result not parametric
Time spent weekly	*Internalising higher*	Confirmed	Confirmed	*Externalising higher*	Confirmed	Confirmed
Social Comparison	*Internalising higher*	Confirmed	Confirmed	*No differences*	Confirmed	Confirmed
Lack of control over time spent	*No differences*	Not confirmed	Not confirmed	*Externalising higher*	Not confirmed	Confirmed*
Monitoring of feedback	*Internalising higher*	Not confirmed	Not confirmed	*No differences*	Confirmed	Confirmed
Impact of feedback on mood	*Internalising higher*	Confirmed	Confirmed	*No differences*	Not confirmed	Not confirmed
Happy about friends	*Internalising lower*	Confirmed	Confirmed	*Externalising lower*	Not confirmed	Confirmed* (but borderline; p = 0.05)
Honest self-disclosure	*Internalising lower*	Confirmed	Confirmed	*No differences*	Confirmed	Confirmed
Authentic self-presentation	*Internalising lower*	Not confirmed	Not confirmed	*No differences*	Confirmed	Confirmed

Note.* denotes tests where we found a discrepancy. The hypotheses preregistered in the Stage 1 protocol are reported in italics.

Supplementary Table 14.

Summary of discrepancies between pre-registered parametric tests and non-parametric test results for Question 3.

Question 3: Internalising/Externalising vs no condition

	**H3: Adolescents with internalising vs externalising conditions**		
Social Media	Hypothesis	Result	Result not parametric
Time spent weekly	*No differences*	Not confirmed	Not confirmed
Social Comparison	*Internalising higher*	Confirmed	Not confirmed*
Lack of control over time spent	*Internalising lower*	Not confirmed	Not confirmed
Monitoring of feedback	*Internalising higher*	Not confirmed	Not confirmed
Impact of feedback on mood	*Internalising higher*	Not confirmed	Not confirmed
Happy about friends	*No differences*	Not confirmed	Not confirmed
Honest self-disclosure	*Internalising lower*	Not confirmed	Not confirmed
Authentic self-presentation	*Internalising lower*	Not confirmed	Not confirmed

Note.* denotes tests where we found a discrepancy. The hypotheses preregistered in the Stage 1 protocol are reported in italics.

Sensitivity analyses

Between group comorbidity

We removed participants with between-group comorbidities (N = 57; i.e., those who received both internalising and externalising diagnoses) when testing Question 2 and 3 for two reasons: 1) our interest in comparing individuals with different core mental health concerns (i.e., internalising vs externalising) required that we remove individuals who belong to both groups, and 2) the impossibility to establish a “primary” diagnosis. The majority of adolescents in this group were diagnosed with a behavioural disorder (N = 48), while a minority had an attention-related disorder (N = 15). The most common comorbid internalising diagnoses were anxiety-related (N = 45) and depression-related (N = 28). Below, we report sensitivity analyses including them.

Supplementary Table 15.

Sensitivity analyses for Question 2 including adolescents with internalising and between-group comorbidities vs no condition.

	**NHST (lm)**				**Equivalence testing (bootstrapped t-TOST)**				**Result**
Social Media	beta	Std. Error (lm)	t value (lm)	p (lm)	g	90%CI lower bound	90%CI higher bound	Equivalence test	Summary
Time spent weekly	1.248	0.099	12.667	6.807E-35	0.66	0.56	0.76	The equivalence test was non-significant, t(386.64) = 4.41, p = 1e+00	Significant and non-equivalent*
Social Comparison	0.708	0.077	9.173	8.301E-20	0.50	0.40	0.61	The equivalence test was non-significant, t(392.92) = 1.677, p = 9.69e-01	Significant and non-equivalent*
Lack of control over time spent	0.585	0.083	7.064	1.988E-12	0.41	0.31	0.52	The equivalence test was non-significant, t(412.88) = 0.240, p = 6.11e-01	Significant and non-equivalent*
Monitoring of feedback	0.178	0.087	2.044	0.041	0.12	0.02	0.22	The equivalence test was significant, t(402.28) = -4.736, p = 0e+00	Not significant and equivalent
Impact of feedback on mood	0.493	0.070	6.997	3.217E-12	0.37	0.27	0.48	The equivalence test was non-significant, t(378.58) = -0.426, p = 3.27e-01	Significant and non-equivalent*
Happy about friends	-0.370	0.047	-7.788	9.276E-15	-0.41	-0.50	-0.31	The equivalence test was non-significant, t(371.28) = -0.130, p = 5.74e-01	Significant and non-equivalent*
Honest self-disclosure	-0.444	0.073	-6.057	1.565E-09	-0.34	-0.44	-0.24	The equivalence test was non-significant, t(384.27) = 1.038, p = 1.39e-01	Significant and non-equivalent*

Authentic self-presentation	-0.218	0.073	-2.991	0.003	-0.17	-0.27	-0.07	The equivalence test was significant, t(380.15) = 3.716, p = 5e-04	Significant and equivalent

*Note***.** Contrast for beta: Internalising + between-group comorbidity (Internalising and Externalising) vs no diagnosis. Summary: * denotes results where we find significant and meaningful differences.

Supplementary Table 16.

Sensitivity analyses for Question 2 including adolescents with externalising and between-group comorbidities vs no condition.

**Social media**	**NHST (lm)**				**Equivalence testing (bootstrapped t-TOST)**				
	Beta (lm)	Std. Error (lm)	t value (lm)	p (lm)	g	90%CI lower bound	90%CI higher bound	Equivalence test	Summary
Time spent weekly	1.036	0.138	7.504	8.180E-13	0.51	0.37	0.65	The equivalence test was non-significant, t(167.41) = 1.206, p = 9.13e-01	Significant and non-equivalent*
Social Comparison	0.075	0.108	0.693	0.488	0.06	-0.08	0.20	The equivalence test was significant, t(168.01) = -4.047, p = 0e+00	Not significant and equivalent
Lack of control over time spent	0.349	0.120	2.901	0.004	0.24	0.09	0.39	The equivalence test was significant, t(162.08) = -1.858, p = 3.35e-02	Significant and equivalent
Monitoring of feedback	0.105	0.125	0.837	0.403	0.07	-0.08	0.22	The equivalence test was significant, t(160.85) = -3.817, p = 0e+00	Not significant and equivalent
Impact of feedback on mood	0.374	0.100	3.760	2.00E-3	0.29	0.15	0.43	The equivalence test was non-significant, t(158.9) = -1.179, p = 1.21e-01	Significant and not-equivalent*
Happy about friends	-0.147	0.067	-2.188	0.029	-0.18	-0.31	-0.03	The equivalence test was significant, t(155.55) = 2.481, p = 1.3e-02	Not significant and not-equivalent
Honest self-disclosure	-0.393	0.104	-3.760	1.732E-3	-0.30	-0.44	-0.16	The equivalence test was non-significant, t(158.56) = 1.131, p = 1.18e-01	Significant and not-equivalent*

Authentic self-presentation	-0.093	0.104	-0.892	0.373	-0.08	-0.21	0.07	The equivalence test was significant, t(157.26) = 3.736, p = 0e+00	Not-significant and equivalent

Note. Beta (lm) contrast: Externalising + between-group comorbidity (Externalising and Internalising) vs no diagnosis. Summary: the * denotes results where we find significant and meaningful differences. Alpha for NHST corrected for multiple comparisons; α = 0.0125.

Mental health severity

Supplementary Table 17.

Sensitivity analysis for mental health severity (number of diagnoses) in relation to each social media item.

Social Media	Term	Estimate (lm)	Std.Error (lm)	t statistic (lm)	P.value (lm)
Time spent weekly	(Intercept)	2.76	0.089	30.75	2.00E-16
	Diagnoses_count	0.51	0.089	5.72	1.78E-08*
Social Comparison	(Intercept)	2.688	0.066	40.494	4.60e-159
	Diagnoses_count	0.214	0.067	3.205	0.001*
Lack of control over time spent	(Intercept)	3.049	0.070	43.678	1.63E-169
	Diagnoses_count	0.076	0.070	1.088	0.277
Monitoring of feedback	(Intercept)	3.558	0.065	54.933	4.76E-212
	Diagnoses_count	0.214	0.065	3.291	0.001*
Impact of feedback on mood	(Intercept)	2.207	0.064	34.422	3.48E-132
	Diagnoses_count	0.163	0.063	2.562	0.011*
Happy about friends	(Intercept)	4.259	0.046	93.448	1.53e-310
	Diagnoses_count	-0.067	0.046	-1.469	0.143
Honest self-disclosure	(Intercept)	3.562	0.061	58.299	4.52E-218
	Diagnoses_count	0.067	0.061	1.107	0.269
Authentic self-presentation	(Intercept)	3.251	0.063	51.724	4.44E-199
	Diagnoses_count	-0.109	0.063	-1.730	0.0843

Note. The * in P.value denotes significance. Further, the predictor (diagnoses_count) refers to the number of diagnoses irrespective of diagnostic category. Alpha for NHST corrected for multiple comparisons; α = 0.0125.

Individual diagnoses

Supplementary Table 18.

Sensitivity analyses for major depressive disorder (MDD; N ≈ 86) vs no condition.

	**NHST (lm)**				**Equivalence testing (bootstrapped t-TOST)**				
Social Media	Beta (lm)	Std. Error (lm)	t value (lm)	p (lm)	g	90%CI lower bound	90%CI higher bound	Equivalence test	Summary
Time spent weekly	1.92	0.18	10.48	2.962E-25	1.04	0.86	1.25	The equivalence test was non-significant, t(87.61) = 5.33, p = 1e+00	Significant and non-equivalent*
Social Comparison	0.87	0.14	6.12	1.09E-09	0.64	0.44	0.85	The equivalence test was non-significant, t(89.54) = 2.093, p = 9.93e-01	Significant and non-equivalent*
Lack of control over time spent	0.81	0.16	5.19	2.28E-07	0.59	0.41	0.80	The equivalence test was non-significant, t(91.69) = 1.869, p = 9.79e-01	Significant and non-equivalent*
Monitoring of feedback	0.06	0.16	0.39	0.694	0.04	-0.15	0.24	The equivalence test was significant, t(88.9) = -3.172, p = 1e-03	Not significant and equivalent
Impact of feedback on mood	0.41	0.13	3.14	0.002	0.31	0.12	0.51	The equivalence test was non-significant, t(87.68) = -0.718, p = 2.36e-01	Significant and non-equivalent*
Happy about friends	-0.37	0.09	-4.19	2.88E-05	-0.42	-0.59	-0.24	The equivalence test was non-significant, t(86.42) = -0.141, p = 5.68e-01	Significant and non-equivalent*
Honest self-disclosure	-0.47	0.14	-3.42	0.001	-0.35	-0.54	-0.16	The equivalence test was non-significant, t(86.93) = 0.413, p = 3.54e-01	Significant and non-equivalent*
Authentic self-presentation	-0.02	0.13	-0.17	0.866	-0.02	-0.19	0.16	The equivalence test was significant, t(87.39) = 3.429, p = 1.5e-03	Not significant and equivalent

*Note***.** Contrast for beta: MDD vs no diagnosis. Alpha for NHST corrected for multiple comparisons; α = 0.0125. The * in Summary denotes results where we find significant and meaningful differences.

Supplementary Table 19.

Sensitivity analyses for generalised anxiety disorder (GAD; N ≈ 77) vs no condition.

	NHST (lm)				Equivalence testing (bootstrapped t-TOST)				
Social Media	Beta (lm)	Std. Error (lm)	t value (lm)	p (lm)	g	90%CI lower bound	90%CI higher bound	Equivalence test	Summary
Time spent weekly	1.54	0.19	8.01	1.7022E-15	0.81	0.61	1.01	The equivalence test was non-significant, t(78.62) = 3.180, p = 1e+00	Significant and not-equivalent*
Social Comparison	0.65	0.16	4.20	2.74E-05	0.44	0.23	0.66	The equivalence test was non-significant, t(74.56) = 0.350, p = 6.44e-01	Significant and not-equivalent*
Lack of control over time spent	0.69	0.17	4.19	2.89E-05	0.51	0.32	0.71	The equivalence test was non-significant, t(79.03) = 1.010, p = 8.77e-01	Significant and not-equivalent*
Monitoring of feedback	0.09	0.17	0.52	0.601	0.06	-0.14	0.26	The equivalence test was significant, t(76.92) = -2.846, p = 3.5e-03	Not significant and equivalent
Impact of feedback on mood	0.58	0.14	4.25	2.17E-05	0.45	0.25	0.65	The equivalence test was non-significant, t(76.88) = 0.416, p = 6.85e-01	Significant and not-equivalent*
Happy about friends	-0.62	0.09	-6.53	7.64E-11	-0.66	-0.88	-0.46	The equivalence test was non-significant, t(72.02) = -1.968, p = 9.87e-01	Significant and not-equivalent*
Honest self-disclosure	-0.46	0.15	-3.15	0.002	-0.33	-0.54	-0.13	The equivalence test was non-significant, t(73.42) = 0.513, p = 2.98e-01	Significant and not-equivalent*
Authentic self-presentation	-0.33	0.14	-2.30	0.021	-0.26	-0.46	-0.06	The equivalence test was non-significant, t(74.22) = 1.107, p = 1.26e-01	Not significant and not-equivalent

*Note***.** Contrast for beta: GAD vs no diagnosis. Alpha for NHST corrected for multiple comparisons; α = 0.0125. The * in Summary denotes meaningful differences.

Supplementary Table 20.
Sensitivity analyses for social anxiety disorder (SAD; N ≈ 71) vs no condition.

	NHST (lm)				Equivalence testing (bootstrapped t-TOST)				
Social Media	Beta (lm)	Std. Error (lm)	t value (lm)	p (lm)	g	90%CI lower bound	90%CI higher bound	Equivalence test	Summary
Time spent weekly	1.56	0.20	7.84	6.357E-15	0.85	0.66	1.07	The equivalence test was non-significant, t(72.55) = 3.510, p = 1e+00	Significant and not-equivalent*
Social Comparison	1.02	0.16	6.45	1.31E-10	0.72	0.51	0.97	The equivalence test was non-significant, t(71.67) = 2.496, p = 9.98e-01	Significant and not-equivalent*
Lack of control over time spent	0.91	0.17	5.32	1.11E-07	0.66	0.43	0.93	The equivalence test was non-significant, t(73.05) = 2.232, p = 9.9e-01	Significant and not-equivalent*
Monitoring of feedback	0.12	0.18	0.68	0.498	0.08	-0.12	0.29	The equivalence test was significant, t(72.44) = -2.597, p = 4.5e-03	Not significant and equivalent
Impact of feedback on mood	0.52	0.14	3.63	0.29E-03	0.40	0.20	0.60	The equivalence test was non-significant, t(70.44) = 0.0201, p = 5.18e-01	Significant and not-equivalent*
Happy about friends	-0.43	0.10	-4.50	7.02E-06	-0.48	-0.68	-0.26	The equivalence test was non-significant, t(70.09) = -0.581, p = 7.44e-01	Significant and not-equivalent*
Honest self-disclosure	-0.37	0.15	-2.42	0.016	-0.27	-0.49	-0.06	The equivalence test was non-significant, t(69.36) = 0.985, p = 1.51e-01	Not significant and not-equivalent
Authentic self-presentation	-0.09	0.15	-0.60	0.550	-0.07	-0.27	0.14	The equivalence test was significant, t(68.97) = 2.568, p = 9e-03	Not significant and equivalent

Note. Contrast: SAD vs no diagnosis. Alpha for NHST corrected for multiple comparisons; α = 0.0125. The * in Summary denotes meaningful differences.

Time spent on school days vs weekends

Supplementary Table 21.

H1: Adolescents with vs without mental health conditions										
	NHST (lm)				Equivalence testing (bootstrapped t-TOST)					
Social Media	Beta (lm)	Std. Error (lm)	t value (lm)	p (lm)	g	90%CI lower bound	90%CI higher bound	Equivalence test	Summary	
Time spent weekdays	0.79	0.08	9.85	1.38E-22	0.43	0.35	0.51	The equivalence test was non-significant, t(624.97) = 0.526, p = 7.15e-01	Significant and not-equivalent*	
Time spent weekends/ holidays	1.07	0.11	10.09	1.39E-23	0.46	0.37	0.54	The equivalence test was non-significant, t(660.4) = 1.126, p = 8.79e-01	Significant and not-equivalent*	
**H2: Adolescents with internalising vs no mental health conditions**										
Time spent weekdays	1.13	0.11	10.53	1.60E-25	0.57	0.46	0.67	The equivalence test was non-significant, t(314.59) = 2.426, p = 9.96e-01	Significant and not-equivalent*	
Time spent weekends/ holidays	1.41	0.14	10.27	2.35E-24	0.61	0.51	0.72	The equivalence test was non-significant, t(324.84) = 3.257, p = 9.99e-01	Significant and not-equivalent*	
**H2: Adolescents with externalising vs no mental health conditions**										
Time spent weekdays	0.58	0.17	3.40	0.683E-3	0.29	0.13	0.46	The equivalence test was non-significant, t(105.37) = -0.981, p = 1.64e-01	Significant and not-equivalent*	
Time spent weekends/ holidays	0.71	0.22	3.22	0.0013	0.30	0.12	0.47	The equivalence test was non-significant, t(107.49) = -0.931, p = 1.73e-01	Significant and not-equivalent*	
**H3: Adolescents with internalising vs externalising mental health conditions**										
Time spent weekdays	0.83	0.08	9.87	1.17E-22	0.23	0.04	0.44	The equivalence test was non-significant, t(172.89) = -1.445, p = 5.84e-02	Significant and not-equivalent*	
Time spent weekends/ holidays	1.10	0.11	10.05	2.05E-23	0.28	0.093	0.48	The equivalence test was non-significant, t(174.63) = -1.004, p = 1.37e-01	Significant and not-equivalent*	

Note. The * in Summary denotes meaningful differences.

Power sensitivity analysis

In addition to our a-priori power calculations, we conducted power sensitivity analyses with the final sample size. That is, we determined the smallest effect size that is observable with 95% power, given an alpha of 0.0125 and our final sample size (Supplementary Table 18). Below we provide conservative estimates based on the social media variables with the smallest sample size.

Supplementary Table 22.

Power sensitivity analyses

Hypothesis	Power sensitivity analysis result
Any condition vs no condition	The smallest effect size detectable was d =|0.19| based on the corrected α = 0.0125 and the overall sample size for adolescents with any mental health condition (N = 519) versus no condition (N= 2821).<br><br>Given that each social media item had a different number of missing values, we also calculated the smallest effect size detectable based on the corrected α = 0.0125 and the sample size for the social media item with the highest number of missing values (authentic self-presentation: N = 475 for adolescents with any condition and N= 2602 for adolescents with no condition), which remained d =|0.19|.
Internalising vs no condition	The smallest effect size detectable was d =|0.24| based on the corrected α = 0.0125 and the overall sample size among adolescents with any internalising condition (N = 282) versus no condition (N = 2821).<br><br>Given that each social media item had a different number of missing values, we also calculated the smallest effect size detectable based on the corrected α = 0.0125 and the sample size for the social media item with the highest number of missing values (authentic self-presentation: N = 267 for adolescents with any condition and N= 2602 for adolescents with no condition), which was d =|0.25|.
Externalising vs no condition	The smallest effect size detectable was d =|0.40| based on the corrected α = 0.0125 and the overall sample size among adolescents with any externalising condition (N = 104) versus no condition (N = 2821).<br><br>Given that each social media item had a different number of missing values, we also calculated the smallest effect size detectable based on the corrected α = 0.0125 and the sample size for the social media item with the highest number of missing values (happy about online friends: N = 91 for adolescents with any externalising condition and N= 2714 for adolescents with no condition), which was d =|0.41|. For this hypothesis, the pre-registered SESOI (d = 0.4) was detectable with 93% power.

Internalising vs externalising condition	The smallest effect size detectable was *d* =|0.45| based on the corrected *α* = 0.0125 and the overall sample size among adolescents with any internalising condition (N = 282) versus externalising condition (N = 104).<br><br>Given that each social media item had a different number of missing values, we also calculated the smallest effect size detectable based on the corrected *α* = 0.0125 and the sample size for the social media item with the highest number of missing values (happy about online friends: N= 272 for adolescents with any internalising condition and N = 91 for adolescents with any externalising condition), which was *d* =|0.47|. For this hypothesis, the pre-registered SESOI (*d* = 0.4) was detectable with 87% power.

Note. OSF code at: https://osf.io/s2dwu/?view_only=5acced2ddb884f9481d439a7746f4dd1

References

1. NHS Digital. MHCYP 2017. https://files.digital.nhs.uk/20/1DEB0D/MHCYP%202017%20Appendix%20F%20-%20Project%20Instructions.pdf.

2. SDQ. https://www.sdqinfo.org/a0.html.

3. DAWBA. https://dawba.info/.

4. Crone, D., Smith, A. & Gough, B. 'I feel totally at one, totally alive and totally happy': a psycho-social explanation of the physical activity and mental health relationship. *Health Education Research* **20**, 600–611 (2005).

5. Knowlden, A. P., Sharma, M. & Bernard, A. L. A Theory of Planned Behavior Research Model for Predicting the Sleep Intentions and Behaviors of Undergraduate College Students. *The Journal of Primary Prevention* **33**, 19–31 (2012).

6. Rebar, A. L. & Taylor, A. Physical activity and mental health; it is more than just a prescription. *Mental Health and Physical Activity* **13**, 77–82 (2017).

7. Mollayeva, T. *et al.* The Pittsburgh sleep quality index as a screening tool for sleep dysfunction in clinical and non-clinical samples: A systematic review and meta-analysis. *Sleep Medicine Reviews* **25**, 52–73 (2016).

8. Ruscio, J. A probability-based measure of effect size: Robustness to base rates and other factors. *Psychological Methods* **13**, 19–30 (2008).

9. Burn-Murdoch, J. Smartphones and social media are destroying children's mental health. Financial Times. https://www.ft.com/content/0e2f6f8e-bb03-4fa7-8864-f48f576167d2 (2023).

10. Haidt, J. Social media is a major cause of the mental illness epidemic in teen girls. Here's the evidence. After Babel. https://jonathanhaidt.substack.com/p/social-media-mental-illness-epidemic (2023).

11. Odgers, C. & Jensen, M. Annual Research Review: Adolescent mental health in the digital age: facts, fears, and future directions. *JOURNAL OF CHILD PSYCHOLOGY AND PSYCHIATRY* **61**, 336–348 (2020).

12. Parry, D. A., Fisher, J. T., Mieczkowski, H., Sewall, C. J. R. & Davidson, B. I. Social Media and Well-being: A Methodological Perspective. *Current Opinion in Psychology* S2352250X21002293 (2021) doi:10.1016/j.copsyc.2021.11.005.

13. Fassi, L. *et al.* Social Media Use and Internalizing Symptoms in Clinical and Community Adolescent Samples: A Systematic Review and Meta-Analysis. *JAMA Pediatr* (2024) doi:10.1001/jamapediatrics.2024.2078.

14. Okoro, C. A. *et al.* Physical Activity Patterns among U.S. Adults with and without Serious Psychological Distress. *Public Health Rep* **129**, 30–38 (2014).

15. Norman, G. R., Sloan, J. A. & Wyrwich, K. W. Interpretation of Changes in Health-related Quality of Life: The Remarkable Universality of Half a Standard Deviation. *Medical Care* **41**, 582–592 (2003).

16. Miller, G. A. The magical number seven plus or minus two: some limits on our capacity for processing information. *Psychol Rev* **63**, 81–97 (1956).

17. Lipsey, M. & Hurley, S. Design Sensitivity: Statistical Power for Applied Experimental Research. in *The SAGE Handbook of Applied Social Research Methods* 44–76 (SAGE Publications, Inc., 2455 Teller Road, Thousand Oaks California 91320 United States, 2009). doi:10.4135/9781483348858.n2.

18. Ferguson, C. J. An effect size primer: A guide for clinicians and researchers. *Professional Psychology: Research and Practice* **40**, 532–538 (2009).

19. Przybylski, A. K., Orben, A. & Weinstein, N. How Much Is Too Much? Examining the Relationship Between Digital Screen Engagement and Psychosocial Functioning in a Confirmatory Cohort Study. *Journal of the American Academy of Child & Adolescent Psychiatry* **59**, 1080–1088 (2020).

20. Jacobson, N. S., & Truax, P. Clinical significance : A statistical approach to defining meaningful change in psychotherapy research. (1992) doi:https://psycnet.apa.org/doi/10.1037/10109-042.

21. Guyatt, G.H., Feeny, P., & Patrick, D.L. Measuring Health-Related Quality of Life. (1993) doi:https://doi.org/10.7326/0003-4819-118-8-199304150-00009.

22. Olatunji, B. O., Cisler, J. M. & Tolin, D. F. Quality of life in the anxiety disorders: A meta-analytic review. *Clinical Psychology Review* **27**, 572–581 (2007).

23. Hampel, P. & Desman, C. [Coping and quality of life among children and adolescents with attention deficit/hyperactivity disorder]. *Prax Kinderpsychol Kinderpsychiatr* **55**, 425–443 (2006).

24. Varni, J. W. & Burwinkle, T. M. The PedsQL™ as a patient-reported outcome in children and adolescents with Attention-Deficit/Hyperactivity Disorder: a population-based study. *Health Qual Life Outcomes* **4**, 26 (2006).

25. Kessler, R. C. *et al.* Patterns and Predictors of Attention-Deficit/Hyperactivity Disorder Persistence into Adulthood: Results from the National Comorbidity Survey Replication. *Biological Psychiatry* **57**, 1442–1451 (2005).
