# Supplementary material for: Social media use in adolescents with and without mental health conditions
Source: Nat Hum Behav. 2025 May 5;9(6):1283–99. doi: 10.1038/s41562-025-02134-4 (PMC12185317; doi:10.1038/s41562-025-02134-4)
Supplement: Supplementary file 2 — Reporting Summary [file 41562_2025_2134_MOESM2_ESM.pdf]

nature portfolio

Corresponding author(s): Fassi Luisa

Last updated by author(s): Jan 27, 2025

Reporting Summary

Nature Portfolio wishes to improve the reproducibility of the work that we publish. This form provides structure for consistency and transparency in reporting. For further information on Nature Portfolio policies, see our Editorial Policies and the Editorial Policy Checklist.

Statistics

For all statistical analyses, confirm that the following items are present in the figure legend, table legend, main text, or Methods section.

n/a	Confirmed	
☐	☒	The exact sample size (n) for each experimental group/condition, given as a discrete number and unit of measurement
☐	☒	A statement on whether measurements were taken from distinct samples or whether the same sample was measured repeatedly
☐	☒	The statistical test(s) used AND whether they are one- or two-sided<br>*Only common tests should be described solely by name; describe more complex techniques in the Methods section.*
☐	☒	A description of all covariates tested
☐	☒	A description of any assumptions or corrections, such as tests of normality and adjustment for multiple comparisons
☐	☒	A full description of the statistical parameters including central tendency (e.g. means) or other basic estimates (e.g. regression coefficient) AND variation (e.g. standard deviation) or associated estimates of uncertainty (e.g. confidence intervals)
☐	☒	For null hypothesis testing, the test statistic (e.g. F, t, r) with confidence intervals, effect sizes, degrees of freedom and P value noted<br>*Give P values as exact values whenever suitable.*
☒	☐	For Bayesian analysis, information on the choice of priors and Markov chain Monte Carlo settings
☒	☐	For hierarchical and complex designs, identification of the appropriate level for tests and full reporting of outcomes
☐	☒	Estimates of effect sizes (e.g. Cohen's d, Pearson's r), indicating how they were calculated

Our web collection on statistics for biologists contains articles on many of the points above.

Software and code

Policy information about availability of computer code

Data collection	This study uses secondary data, details on how to access the data are reported in the Data Availability Statement. Specifically, the MHCYP dataset is held on behalf of NHS Digital by the UK Data Service. Restrictions apply to the availability of this data for privacy and ethical reasons, which were used under license for this study. Data access can be requested by applying to the Data Access Request Service (DARS; number for this study: DARS-NIC-424336-T7K7T-v0.6). Researchers interested in accessing the data can find further information on the DARS website (see https://digital.nhs.uk/services/data-access-request-service-dars/dars-guidance).
Data analysis	The analysis code used to run the power analysis (stage 1) and all other analyses (stage 2) can be found at: https://osf.io/s2dwu/?view_only=5acced2ddb884f9481d439a7746f4dd1. Details on each code script are reported in the READ.ME file.<br><br>We used R version 4.4.0 (2024-04-24), and the following packages: Cairo (version: 1.6-2), readstata13 (version: 0.10.1), dplyr (version 1.1.2), TOSTER (version: 0.8.0), ggplot2 (version 3.5.1), patchwork (version: 1.2.0.9000), tidyverse (version: 2.0.0).

For manuscripts utilizing custom algorithms or software that are central to the research but not yet described in published literature, software must be made available to editors and reviewers. We strongly encourage code deposition in a community repository (e.g. GitHub). See the Nature Portfolio guidelines for submitting code & software for further information.

Data

Policy information about availability of data

All manuscripts must include a data availability statement. This statement should provide the following information, where applicable:

- Accession codes, unique identifiers, or web links for publicly available datasets
- A description of any restrictions on data availability
- For clinical datasets or third party data, please ensure that the statement adheres to our policy

The MHCYP dataset is held on behalf of NHS Digital by the UK Data Service. Restrictions apply to the availability of this data for privacy and ethical reasons, which were used under license for this study. Data access can be requested by applying to the Data Access Request Service (DARS; number: DARS-NIC-424336-T7K7T-v0.6). Researchers interested in accessing the data can find further information on the DARS website (i.e., https://digital.nhs.uk/services/data-access-request-service-dars/dars-guidance).

Research involving human participants, their data, or biological material

Policy information about studies with human participants or human data. See also policy information about sex, gender (identity/presentation), and sexual orientation and race, ethnicity and racism.

Reporting on sex and gender

Our final sample included 3,340 young people aged 11 to 19 (age: mean = 14.77, sd = 2.48). The sample was 50% male and 50% female. Both sex and age were measured using self-report. We provide descriptives for age and sex separately for each group in Table 3 and the section 'population characteristics' below. We also provide separate descriptive statistics separately for males and females (sex variable) for each social media item in the Supplementary Results (Supplementary Table 5-8). We do not provide separate descriptive statistics for age as this question falls outside the scope of this study.

Reporting on race, ethnicity, or other socially relevant groupings

While it is common in research to use statistical control to remove confounding effects from a regression coefficient, appropriate control variables should be identified only after justifying a causal structure that includes the outcome, exposure and all theorised confounders. When the selected control variables are inappropriate or remain unjustified, controlling can result in biased regression estimates. Further, recent literature warns against controlling for demographic factors such as sex without thought and instead prompts researchers to interrogate how this variable intersects with the exposure and outcomes under investigation. In the present work, treating sex or age as a confounding variable would mean ignoring the possibility that there are meaningful sex or age differences in the examined relationships. As our goal is to investigate the association between social media use and mental health diagnosis, we provided a descriptive account of the age and sex of adolescents in each group. As shown below, age and sex of adolescents with vs without a mental health condition are reported for descriptive purposes.

Population characteristics

The age and sex of each examined group are reported in Table 3 in the main manuscript and below:

- No mental health (N = 2821, age mean = 14.71, male proportion: 0.50)
- Any mental health condition (N = 519, age mean = 15.10, male proportion = 0.47)
- Externalising condition(N = 104, age mean = 14.27, male proportion = 0.72)
- Internalising condition (N = 282, age mean = 15.94, male proportion = 0.80)
- Other conditions (N = 76, age mean = 14, male proportion = 0.80)
- Comorbidity between internalising and externalising condition (N = 57, age mean = 13.93, male proportion = 0.51).

Recruitment

The MHCYP study is one of a series of national surveys on the mental health of children and young people in England administered in 1999, 2004, 2017, 2021 and 2022. In this Registered Report, we analysed the 2017 wave collected between January and October 2017: the most recent wave to be made available to researchers as well as the first wave to collect comprehensive data on adolescents' social media use and to include 17 to 19-year-olds. We only analysed data from adolescents who reported being social media users aged 11-19 years, a total of 3,340 participants (50 % male, 50% female) out of the full sample of 9,117. The survey was collected using a stratified probability sample of children and young people living in England who were registered with a general practitioner. Data was collected via face-to-face interviews with adolescents and their parents. At the same time, if the family agreed, questionnaires were mailed to teachers (for the available data and key demographics see MHCYP 2017: https://digital.nhs.uk/data-and-information/publications/statistical/mental-health-of-children-and-young-people-in-england/2017/2017).

Ethics oversight

The MHCYP 2017 survey was reviewed and approved by the West London & GTAC Research Ethics Committee (REC reference: 16/LO/0155) and the Health Research Authority Confidentiality Advisory Group (CAG reference: 16/CAG/0016) in 2016. Both parents and children provided consent to take part in data collection and were compensated with a 10£ voucher for their time. Parents of children under 16 years were interviewed first, and permission was sought to interview their child afterwards; the child then provided assent. Conversely, 17–19-year-olds were directly asked for their consent, with permission subsequently sought for their parents to be interviewed.

Note that full information on the approval of the study protocol must also be provided in the manuscript.

Field-specific reporting

Please select the one below that is the best fit for your research. If you are not sure, read the appropriate sections before making your selection.

☐ Life sciences ☒ Behavioural & social sciences ☐ Ecological, evolutionary & environmental sciences

Behavioural & social sciences study design

All studies must disclose on these points even when the disclosure is negative.

Study description	This study analyses data from a quantitative cross-sectional survey collected in 2017 (Mental Health of Children and Young People).
Research sample	For the 2017 survey, a stratified, multistage random probability sample of 18,029 children was drawn from the NHS Patient Register in October 2016. Children and young people were eligible to participate if they were aged 2 to 19, lived in England, and were registered with a GP. The sample was designed to be representative of the population of children and young people aged 2 to 19 living in England. The final sample consisted of 9,117 children and young people. For this study, we analyzed data from 3,340 participants. This subset was selected based on age (focusing on adolescents aged 9–11 years) and social media use (including only those who reported being social media users).
Sampling strategy	A stratified multistage random probability sample was used for the survey, involving a two-stage process. Full information on the sampling can be found at: https://files.digital.nhs.uk/60/1CB03A/MHCYP%202017%20Survey%20Design%20and%20Methods.pdf<br><br>To determine the sample size needed to answer our research questions, we run power analyses and power sensitivity analyses. A priori power calculations were conducted and reported in the Stage 1 registered report. For these calculations, we set were the smallest effect size of interest (SESOI, d = 0.4; see Supplementary Methods for details), an alpha level of 0.05, and an estimated sample size of N = 3,854. The code for these calculations is available on the Open Science Framework (OSF) at https://osf.io/s2dwu/?view_only=5acced2ddb884f9481d439a7746f4dd1. Results showed that we were sufficiently powered (> 95%) to detect our smallest effect size of interest for all research questions.<br><br>In addition to the a priori power calculations, we conducted power sensitivity analyses using the final sample size, which was known only after starting data analysis. In this case we determined the smallest effect size that could be detected with 95% power, given an alpha level of 0.0125 (corrected for multiple comparisons) and the final sample size (N = 3340). As reported in Supplementary Table 18, these analyses demonstrated power to detect our smallest effect size of interest (d = 0.4) across our research questions.
Data collection	Data were collected as part of a national survey. Researchers were therefore blind to the study conditions and hypotheses. All interviews were conducted individually, involving only the clinical rater and the interviewee (child, parent, or teacher) and were carried out either in person or in a computerized format.<br><br>For participants aged 11 to 16 years, the process began with an initial interview with the parent or legal guardian, followed by a separate interview with the child. Young people aged 17 to 19 were interviewed directly, with their parent also interviewed if both parties consented. Teachers of 5 to 16-year-olds were invited to complete an online or paper questionnaire if consent was provided.<br><br>Mental health assessments were conducted using the detailed and comprehensive Development and Well-Being Assessment (DAWBA; Goodman et al., 2000), which evaluates a range of mental health conditions, including emotional, hyperactivity, and behavioral disorders, as well as less common conditions like autism. After completing the interviews, trained clinical raters reviewed the data to assess the presence of mental health conditions for each participant.
Timing	Data collection occurred over nine months, from January to September 2017. For participants aged 11–16 years, data collection was conducted between January and June 2017 to maintain consistency with previous surveys in the series and to ensure that teacher questionnaires could be completed and returned before the end of the school summer term. For children aged 16 or younger, data collection began with an interview with the parent. Parental permission was then sought to interview the child. If consent was given, the child participated in an interview, which included a self-completion section for sensitive questions. For young people aged 17–19 years, agreement to participate was obtained directly from the individual. Further details on data collection can be found at: https://files.digital.nhs.uk/60/1CB03A/MHCYP%202017%20Survey%20Design%20and%20Methods.pdf
Data exclusions	In this study, we excluded participants aged 2–10 years (N= 5060) and those who reported not using social media (N = 717). Both these exclusions were pre-established (in our Stage 1 registered report) for two reasons: 1) children younger than 11 are in a different developmental time window (childhood/pre-adolescence), 2) we were interested in young people that used social media to answer our research questions. Each social media use item was analyzed independently, allowing us to retain data for individual items even when responses were incomplete for other items. For example, if a child answered the question about time spent on social media but not the question about online social comparison, their response to the time spent question was still included in the analysis. Consequently, the exact sample size varies for each question and is reported in Supplementary Tables 5–8.
Non-participation	No participant declined participation.
Randomization	We did not perform randomization nor we controlled for third variables. While it is common in research to use statistical control to remove confounding effects from a regression coefficient, appropriate control variables should be identified only after justifying a causal structure that includes the outcome, predictors, and all theorised confounders. When the selected control variables are inappropriate or remain unjustified, controlling can result in biased regression estimates. Further, recent literature warns against controlling for demographic factors such as sex without thought and instead prompts researchers to interrogate how this variable intersects with the predictors and outcomes under investigation. In the present work, treating sex or age as a confounding variable would mean ignoring the possibility that there are meaningful sex or age differences in the examined relationships. As our goal is to investigate the overall association between social media use and mental health diagnosis, we provided a descriptive account of the age and sex of adolescents included in each tested model rather than control for these demographics.

Reporting for specific materials, systems and methods

We require information from authors about some types of materials, experimental systems and methods used in many studies. Here, indicate whether each material, system or method listed is relevant to your study. If you are not sure if a list item applies to your research, read the appropriate section before selecting a response.

Materials & experimental systems

n/a	Involved in the study
☒	☐ Antibodies
☒	☐ Eukaryotic cell lines
☒	☐ Palaeontology and archaeology
☒	☐ Animals and other organisms
☒	☐ Clinical data
☒	☐ Dual use research of concern
☒	☐ Plants

Methods

n/a	Involved in the study
☒	☐ ChIP-seq
☒	☐ Flow cytometry
☒	☐ MRI-based neuroimaging

Plants

Seed stocks	NA
Novel plant genotypes	NA
Authentication	NA
